# Photoprotective Effects of *Dendrobium officinale* Protein Hydrolysate Fractions Against UVB-Induced Photoaging Associated with Modulation of MAPK/NF-κB and TGF-β/Smad Signaling

**DOI:** 10.3390/molecules31121990

**Published:** 2026-06-07

**Authors:** Jinghan Zhang, Yue Sun, Jinhao Zheng, Can Yang, Mingshuo Yang, Liming Pan

**Affiliations:** 1 Key Laboratory of State Administration of Traditional Chinese Medicine for Production & Development of Cantonese Medicinal Materials, Guangzhou 510006, China; m13466126549@163.com (J.Z.); sy15292629878@163.com (Y.S.); hxg73663@163.com (J.Z.); 17819689464@163.com (C.Y.); ymsscience@163.com (M.Y.); 2 School of Traditional Chinese Medicine, Guangdong Pharmaceutical University, Guangzhou 510006, China

**Keywords:** *Dendrobium officinale*, protein hydrolysates, UVB-induced photoaging, oxidative stress, photoprotection, HaCaT cells, functional ingredients

## Abstract

*Dendrobium officinale* has attracted increasing attention as a functional food because of its diverse biological activities; however, the photoprotective potential of its protein-derived peptides remains poorly understood. In this study, *D. officinale* protein hydrolysates were fractionated by ultrafiltration according to molecular weight, and their protective effects against ultraviolet B (UVB)-induced photoaging were systematically evaluated in HaCaT keratinocytes. Among the tested fractions, low-molecular-weight peptide fractions exhibited relatively stronger antioxidant activity and effectively reduced intracellular reactive oxygen species (ROS) accumulation in UVB-irradiated cells. In addition, the peptide fractions alleviated UVB-induced inflammatory responses and decreased matrix metalloproteinase (MMP) expression, which was associated with modulation of mitogen-activated protein kinase (MAPK) and nuclear factor kappa B (NF-κB) signaling pathways. Higher-molecular-weight fractions showed relatively stronger effects on maintaining skin barrier-related functions and were associated with regulation of transforming growth factor-β/Smad (TGF-β/Smad) signaling and collagen-related protein expression. Overall, these findings demonstrate functional differences among *Dendrobium officinale* peptide fractions and suggest their potential application as natural photoprotective ingredients in functional foods and cosmeceutical products.

## 1. Introduction

The skin is the largest organ of the human body and the primary interface with the external environment [1]. Its structural integrity and physiological function rely on coordinated interactions among the epidermis, dermis, and subcutaneous tissue, as well as the extracellular matrix (ECM) network, which maintains skin firmness, elasticity, and barrier function [2,3]. Among various environmental stressors, solar ultraviolet (UV) radiation is considered the dominant cause of extrinsic skin aging. Ultraviolet B (UVB) radiation (280–315 nm), with a higher photon energy than ultraviolet A (UVA), penetrates the epidermis and upper dermis, directly damages nucleic acids and proteins, and promotes excessive production of reactive oxygen species (ROS) [4,5]. ROS attack lipids, proteins, and DNA, activate redox-sensitive signaling pathways such as nuclear factor-κB (NF-κB) and mitogen-activated protein kinases (MAPKs), and induce the overexpression of matrix metalloproteinases (MMPs) and pro-inflammatory cytokines [6,7,8]. Consequently, collagen fibers and other ECM components are degraded, the basement membrane and skin barrier structure are disrupted, and clinical manifestations of photoaging, including wrinkles, skin laxity, and roughness, gradually appear [6,9,10].

The skin possesses endogenous defense systems against UV-induced damage, including enzymatic antioxidants such as superoxide dismutase (SOD), catalase (CAT), and glutathione peroxidase (GSH-Px), as well as non-enzymatic antioxidants [11]. These systems help to maintain redox homeostasis and limit oxidative damage. UV exposure also induces adaptive responses, such as epidermal thickening, melanogenesis, and upregulation of growth factors that support barrier repair [12,13]. However, with chronic or excessive UVB exposure and aging, antioxidant and repair capacities decline, ROS accumulate, DNA damage persists, and signaling cascades such as the Mitogen-Activated Protein Kinase (MAPK)/Activator Protein-1 (AP-1) and Nuclear Factor Kappa B (NF-κB) remain activated [14,15], whereas TGF-β/Smad signaling and collagen synthesis are suppressed, whereas matrix metalloproteinase-1 (MMP-1) and matrix metalloproteinase-3 (MMP-3) are upregulated, further promoting ECM degradation [16,17,18]. This multifactorial pathogenesis suggests that effective anti-photoaging strategies should combine direct antioxidant activity with the modulation of inflammation, matrix remodeling, and barrier function.

*Dendrobium officinale* Kimura et Migo is a high-value medicinal and food homologous herb that is widely used in traditional Chinese medicine [19]. The processed stem “Tiepi Fengdou” is recorded in the Chinese Pharmacopoeia for indications such as nourishing Yin, promoting body fluids, and alleviating deficiency-related symptoms [20,21]. Modern phytochemical and pharmacological studies have focused primarily on polysaccharides, alkaloids, and flavonoids as the main active components. *D. officinale* polysaccharides exhibit prominent antioxidant, immunomodulatory, anti-fatigue, and hypoglycemic activities, partly through free radical scavenging, enhancement of antioxidant enzymes, and regulation of gut microbiota [22,23,24]. Alkaloids and flavonoids contribute additional antioxidant, anti-inflammatory, and anti-tumor effects [25,26,27,28].

In contrast, the protein fraction of *D. officinale* has been studied much less, despite accounting for approximately 3–8% of the dry weight and containing essential amino acids [29]. Proteins from other food and medicinal sources can yield bioactive peptides after enzymatic hydrolysis, with reported antioxidant, anti-inflammatory and tissue-protective properties relevant to skin health and photoaging [30,31]. For *D. officinale*, extraction of protein, optimization of enzymatic hydrolysis and fractionation, and the evaluation of protein-derived peptides in a UVB-induced skin damage model have not been systematically addressed. The anti-photoaging potential and underlying mechanisms of *D. officinale* protein hydrolysates therefore remain largely unknown.

This study addresses this gap by focusing on *D. officinale* protein-derived peptides as potential anti-photoaging agents. The protein extraction and enzymatic hydrolysis conditions were optimized, and ultrafiltration was used to obtain fractions with defined molecular weight ranges. The native protein (TD), total hydrolysate (TDM), and two ultrafiltration fractions (TDM-5, 10–50 kDa; TDM-1, <10 kDa) were prepared and characterized. Their antioxidant properties were evaluated in vitro using multiple radical-scavenging and reduction assays. A UVB-induced HaCaT keratinocyte model was used to assess the cytoprotective effects of the extracts, including cell viability, proliferation, ROS generation, and antioxidant indices (Malondialdehyde (MDA), SOD, and Total Antioxidant Capacity (T-AOC)).

To further elucidate the molecular mechanisms underlying the protective effects of *Dendrobium officinale* Protein Hydrolysates (DOPH) against UVB-induced skin damage, genes associated with epidermal barrier integrity (filaggrin, involucrin, and loricrin), skin hydration (Aquaporin-3 (AQP-3), Caspase-14, and hyaluronan synthase 2 (HAS-2), inflammatory response (*Interleukin-1β (IL-1β), interleukin-6 (IL-6), interleukin-8 (IL-8), and Tumor Necrosis Factor-α (TNF-α*)), and extracellular matrix degradation/photoaging (MMP-1 and MMP-3) were selected for Real-Time Quantitative Polymerase Chain Reaction (RT-qPCR) analysis. These genes are widely recognized as key biomarkers involved in UVB-induced skin barrier dysfunction, dehydration, inflammation, and collagen degradation. Western blotting was used to investigate changes in MAPK/AP-1, NF-κB, and TGF-β/Smad signaling in UVB-exposed HaCaT cells treated with selected peptide fractions. Through this integrated approach, this study aimed to develop and evaluate fractionated protein hydrolysates from *D. officinale* as potential functional ingredients with photoprotective activity.

## 2. Results and Discussion

### 2.1. Optimization and Characterization of Dendrobium officinale Protein Hydrolysates

#### 2.1.1. Effects of Single-Factor Conditions on the Degree of Hydrolysis

**Figure 1:**
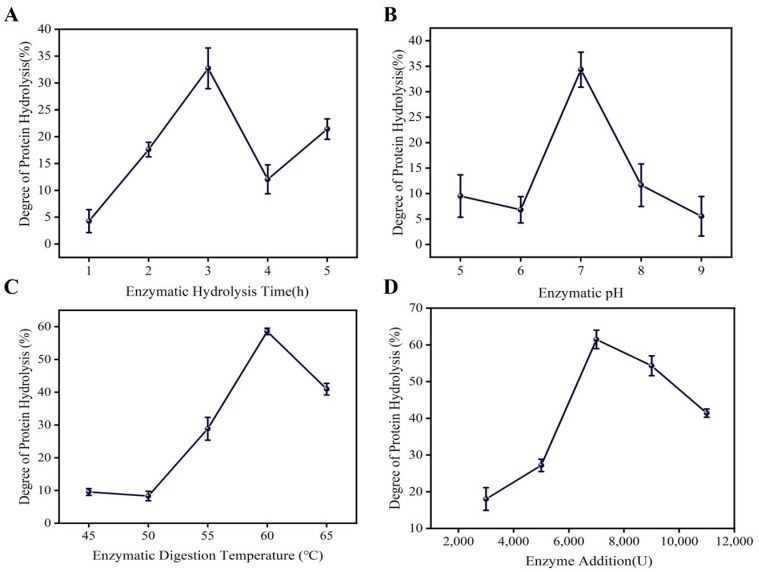
Effects of unifactorial conditions on proteolysis degree of *Dendrobium officinale***.** (**A**): The Effect of Enzymatic Hydrolysis Time on the Degree of Protein Hydrolysis. (**B**): Effect of Reaction pH on Protein Hydrolysis Rate by Enzymatic Means. (**C**): Effect of Temperature on Protein Degradation During Enzymatic Hydrolysis. (**D**): Effect of Varying Enzyme Levels on the Extent of Protein Hydrolysis.

The degree of hydrolysis (DH) of *D. officinale* protein was first examined under different single-factor conditions. With substrate concentration fixed at 0.3% (*w*/*v*), pH 6.9, enzyme dosage 5000 U and temperature 55 °C, extension of hydrolysis time from 1 to 5 h produced a typical bell-shaped curve (Figure 1A). DH increased rapidly during the first 3 h, reached a maximum at 3 h, and then declined slightly thereafter.

The initial rise in DH suggests that readily accessible peptide bonds on the protein surface were rapidly cleaved by neutral protease and papain, leading to efficient generation of peptides. The subsequent decline may be related to gradual enzyme inactivation at prolonged incubation, feedback inhibition by accumulated peptides, or secondary aggregation of released peptides, all of which reduce the apparent number of newly cleavable sites. Based on this pattern, 3 h was selected as the appropriate hydrolysis time for further optimization.

At a fixed substrate concentration of 0.3%, hydrolysis time 3 h, enzyme dosage 5000 U and temperature 55 °C, the influence of pH was examined in the range 5–9 (Figure 1B). DH increased as pH approached neutrality, peaked at pH 7.0, and then decreased at higher pH. This profile aligns with the known optimum of neutral proteases and papain around neutral pH, where the ionization state of catalytic residues and substrate side chains is favorable for catalysis. Under more acidic or alkaline conditions, charged states of key amino acids may shift, weakening enzyme–substrate interaction and lowering catalytic efficiency. Thus pH 7.0 was considered the most suitable hydrolysis pH.

Temperature is another key determinant of enzymatic hydrolysis. At substrate concentration 0.3%, pH 6.9 and enzyme dosage 5000 U, an increase in temperature led to a steady rise in DH, which reached a maximum at 60 °C, followed by a decline at higher temperatures (Figure 1C). Moderate heating accelerates molecular motion and substrate–enzyme collision, enhancing hydrolysis. However, excessive temperature can destabilize protein conformation and accelerate thermal denaturation of proteases. A temperature of 60 °C therefore provided a practical compromise between catalytic rate and enzyme stability and was chosen as the working temperature for further design.

Finally, the effect of enzyme dosage was assessed at substrate concentration 0.3%, pH 6.9 and 55 °C (Figure 1D). As the enzyme dosage increased, DH rose and reached a peak near 7000 U, then slightly decreased at higher dosages. Similar behavior has been reported for other protein substrates, where excessive enzyme can cause non-productive enzyme–enzyme interactions, over-saturation relative to available substrate, or changes in solution viscosity, resulting in decreased catalytic efficiency per unit enzyme. Considering both DH and enzyme cost, 7000 U was selected as a reasonable dosage for optimization.

Overall, the single-factor experiments showed that *D. officinale* protein hydrolysis is governed by clearly defined optimal windows for time, pH, temperature and enzyme dosage, and provided rational center points for subsequent response surface methodology (RSM).

#### 2.1.2. Response Surface Optimization and Model Validation

On the basis of the single-factor results, hydrolysis time (A), enzyme dosage (B) and pH (C) were chosen as independent variables and DH as the response. A Box–Behnken design was employed, and the experimental design and results are listed in Table S5. The data were fitted to a second-order polynomial model using Design-Expert 13, yielding the regression equation:

y = 40.64 − 0.6582A + 0.1113B + 1.32C + 1.21AB − 0.9075AC + 1.47BC − 2.75A^2^ − 5.11B^2^ − 1.57C^2^

ANOVA of the regression is summarized in Table S5. The model was highly significant (*p* < 0.0001), with an F-value of 242.60. The coefficient of determination (R^2^ = 0.9968) and adjusted R^2^ (0.9927) indicated that more than 99% of the variability in DH was explained by the model. The lack-of-fit term was not significant (*p* = 0.0644), confirming that the model adequately described the experimental data without systematic deviation.

Among the linear terms, hydrolysis time (A) and pH (C) significantly affected DH, whereas the direct effect of enzyme dosage (B) was relatively weak. All interaction terms (AB, AC and BC) were significant or highly significant, suggesting that the effect of one factor depends strongly on the levels of the others. The quadratic terms A^2^, B^2^ and C^2^ were all highly significant, which is consistent with the existence of a distinct maximum in the three-dimensional response surface. According to F-values, the relative importance of the three factors followed the order: pH (C) > time (A) > enzyme dosage (B). These results emphasize the primary role of pH in controlling protein hydrolysis, with hydrolysis time as the second most influential factor.

**Figure 2:**
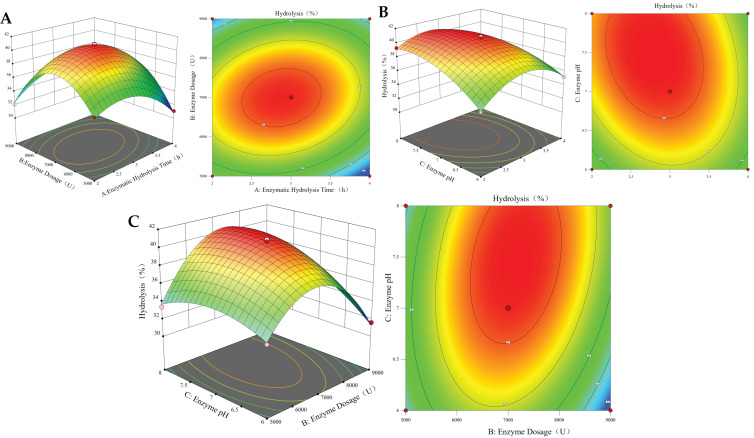
Response Surface Plot of the Degree of Protein Hydrolysis of *Dendrobium officinale*. (**A**): Correlation Between Enzymatic Hydrolysis Time and Enzyme Dosage. (**B**): Correlation Between Enzymatic Hydrolysis Time and pH. (**C**): Correlation Between Enzyme Dosage and Enzymatic Hydrolysis pH. The color gradient from blue to red represents an increasing degree of hydrolysis (%).

Response surface and contour plots (Figure 2) clearly visualized the interactions. The surface describing the interaction between enzyme dosage and pH was the steepest, indicating that small deviations in these two parameters can markedly change DH. This is in agreement with the ANOVA results showing strong AB and BC interaction effects.

The optimization module of the software predicted the following theoretical optimum conditions: hydrolysis time 2.946 h, enzyme dosage 7159 U, and pH 7.09, at which the predicted maximum DH was 40.76%. For practical application and ease of control, the conditions were slightly adjusted to hydrolysis time 3.0 h, enzyme dosage 7100 U and pH 7.10. Under these conditions, three independent validation experiments yielded DH values of 41.65%, 41.77% and 41.74%, all slightly higher than the predicted value and with low variation. This close agreement confirmed that the RSM model is robust and can accurately predict and optimize *D. officinale* protein hydrolysis in practice.

Under the optimized protein extraction conditions (60 °C, 0.1 mol/L extraction solution, 1.5 h extraction time, solid–liquid ratio of 1:55, and acid precipitation at pH 4.0), the total protein content of *D. officinale* dry powder was determined to be 45.50 mg/g, and the extracted protein powder showed a protein purity of 74.81%. The obtained protein samples were subsequently used for enzymatic hydrolysis and ultrafiltration fractionation.

#### 2.1.3. Molecular Weight Distribution of Hydrolysates and Ultrafiltration Fractions

**Figure 3:**
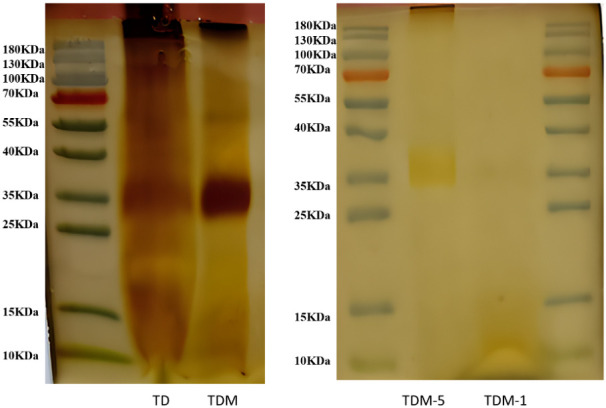
Distribution Profile of Proteins and Ultrafiltered Enzymatic Hydrolysates from *Dendrobium officinale.*

The molecular weight distribution of the native protein (TD), crude enzymatic hydrolysate (TDM) and ultrafiltration fractions (TDM-5, TDM-1) was evaluated by SDS–PAGE (Figure 3). TD displayed distinct bands at approximately 130, 100, 35 and 15 kDa, indicating that the crude extract consisted mainly of medium- and high-molecular-weight proteins.

After combined hydrolysis with neutral protease and papain, TDM showed an obvious shift toward lower molecular masses. Major bands appeared around 100, 55, 35, 25 and 10 kDa, and the pattern became more continuous, reflecting extensive cleavage of large proteins into a heterogeneous mixture of peptides.

Further fractionation of TDM using 50 and 10 kDa cut-off ultrafiltration membranes produced two fractions: TDM-5 (10–50 kDa) and TDM-1 (<10 kDa). TDM-5 retained bands mainly in the 35–40 kDa region and showed a substantial depletion of proteins above 50 kDa. TDM-1 was enriched in peptides around and below 10 kDa, with almost no visible bands in the higher molecular weight region. These data confirm that ultrafiltration can enrich defined molecular weight windows and improve the relative purity of specific peptide size classes, although at the cost of total protein yield.

Peptide size is known to influence antioxidant and cytoprotective activity [32,33]. Smaller peptides often display higher solubility, faster diffusion and better access to cellular or molecular targets. The clear separation of TDM-5 and TDM-1 in this study therefore provides a suitable basis for exploring structure–activity relationships between peptide size distribution and antioxidant or anti-photoaging efficacy in subsequent assays.

### 2.2. In Vitro Antioxidant Activities of D. officinale Protein and Ultrafiltration Fractions

Four complementary in vitro assays were used to characterize the antioxidant capacity of TD, TDM, TDM-5 and TDM-1: DPPH· scavenging, ABTS^+^· scavenging, ferric-reducing antioxidant power (FRAP) and hydroxyl radical (·OH) scavenging.

#### 2.2.1. DPPH Radical Scavenging Activity

**Figure 4:**
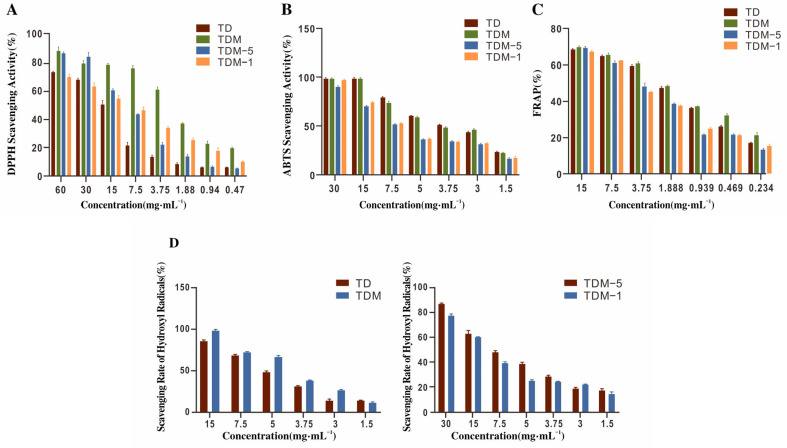
In vitro antioxidant activities of *D. officinale* protein and ultrafiltration fractions. (**A**): DPPH Scavenging Activity of *Dendrobium officinale* Protein and Its Enzymatic Hydrolysis Ultrafiltration Products. (**B**): ABTS Scavenging Activity of *Dendrobium officinale* Protein and Its Enzymatic Hydrolysis Ultrafiltration Products. (**C**): Total Antioxidant Capacity of *Dendrobium officinale* Protein and Its Enzymatic Hydrolysis Ultrafiltration Products. (**D**): Scavenging Rate of Hydroxyl Radicals (OH·) by *Dendrobium officinale* Protein and Enzymatic Hydrolysates Fractions.

As shown in (Figure 4A), all four samples scavenged DPPH radicals in a concentration-dependent manner over 0.47–60 mg·mL^−1^. The IC_50_ values were 10.08 mg·mL^−1^ for TD, 2.772 mg·mL^−1^ for TDM, 9.476 mg·mL^−1^ for TDM-5 and 5.294 mg·mL^−1^ for TDM-1.

The approximately three- to four-fold lower IC_50_ of TDM compared with TD indicates that enzymatic hydrolysis greatly enhanced the DPPH-scavenging capacity of *D. officinale* protein. Enzymatic treatment likely exposed buried aromatic, sulfur-containing and hydrophobic amino acid residues, which can donate hydrogen atoms or electrons to neutralize DPPH radicals. Among the ultrafiltration fractions, TDM-1 showed stronger DPPH-scavenging activity than TDM-5, consistent with the notion that lower-molecular-weight peptides possess higher radical-scavenging capacity, possibly because of improved mass transfer and increased accessibility to DPPH in solution.

#### 2.2.2. ABTS Radical Scavenging Activity

For ABTS^+^·, all samples again showed dose-dependent scavenging behavior in the 1.5–30 mg·mL^−1^ range (Figure 4B). The IC_50_ values were 4.743 mg·mL^−1^ (TD), 4.591 mg·mL^−1^ (TDM), 18.68 mg·mL^−1^ (TDM-5) and 18.14 mg·mL^−1^ (TDM-1).

TDM displayed a slightly lower IC_50_ than TD, suggesting a modest improvement in ABTS^+^· scavenging following hydrolysis. However, both ultrafiltration fractions exhibited markedly higher IC_50_ values than TDM, indicating weakened activity when the hydrolysate was subdivided into narrower molecular weight ranges. ABTS scavenging involves predominantly electron-transfer reactions with a contribution from hydrogen atom transfer. The weaker performance of TDM-5 and TDM-1 suggests that synergistic effects among peptides of various sizes in the unfractionated hydrolysate contribute to ABTS scavenging, and that fractionation can disrupt such synergy.

#### 2.2.3. Ferric-Reducing Antioxidant Power (FRAP)

In the FRAP assay, the capacity of each sample to reduce Fe^3+^ to Fe^2+^ increased with rising concentration from 0.234 to 15 mg·mL^−1^ (Figure 4C). The IC_50_ values were 1.313 mg·mL^−1^ for TD, 1.137 mg·mL^−1^ for TDM, 3.043 mg·mL^−1^ for TDM-5 and 3.319 mg·mL^−1^ for TDM-1.

TD and TDM showed no substantial difference in FRAP activity, indicating that the overall electron-donating ability of *D. officinale* protein was only slightly affected by hydrolysis. In contrast, TDM-5 and TDM-1 exhibited lower reducing capacity than TDM, again suggesting that the presence of a broad spectrum of peptide sizes is advantageous for sustaining total reducing power. Removal of certain peptide classes during ultrafiltration may weaken the collective electron-donating potential.

#### 2.2.4. Hydroxyl Radical Scavenging Activity

Hydroxyl radicals are highly reactive and are closely associated with lipid peroxidation and DNA damage. As illustrated in (Figure 4D), TD, TDM, TDM-5 and TDM-1 all scavenged ·OH in a dose-dependent fashion over 1.5–30 mg·mL^−1^. The IC_50_ values were 5.118 mg·mL^−1^ (TD), 4.586 mg·mL^−1^ (TDM), 14.89 mg·mL^−1^ (TDM-5) and 11.75 mg·mL^−1^ (TDM-1).

Compared with TD, TDM showed a slightly lower IC_50_, indicating improved hydroxyl radical scavenging after hydrolysis. Among the ultrafiltration fractions, TDM-1 again outperformed TDM-5, in line with the DPPH results, supporting the view that low-molecular-weight peptides are more effective in scavenging aggressive radicals. These peptides may either quench ·OH or chelate transition metals that catalyze its formation.

#### 2.2.5. Comparative Analysis of Antioxidant Profiles

Taken together, the in vitro antioxidant data demonstrate that enzymatic hydrolysis markedly improves the radical-scavenging capacity of *D. officinale* protein, especially in the DPPH and hydroxyl radical systems. TDM showed the strongest overall performance in DPPH and ABTS assays and retained good FRAP activity, implying that a broad, unfractionated distribution of peptide sizes is favorable for multi-mechanistic radical scavenging.

The <10 kDa fraction TDM-1 exhibited particularly strong DPPH and ·OH scavenging, highlighting the advantage of small peptides for neutralizing highly reactive species, whereas its performance in ABTS and FRAP was relatively weaker. TDM-5 showed intermediate activity between TDM and TDM-1. These differentiated antioxidant profiles suggest that each fraction may contribute to photoprotection associated with distinct but complementary mechanisms, and provide a rationale for further evaluation in UVB-induced HaCaT cell models.

### 2.3. Protective Effects of Hydrolysate Fractions on UVB-Induced HaCaT Cell Damage

#### 2.3.1. Cytotoxicity Evaluation of TD, TDM, TDM-5 and TDM-1

**Figure 5:**
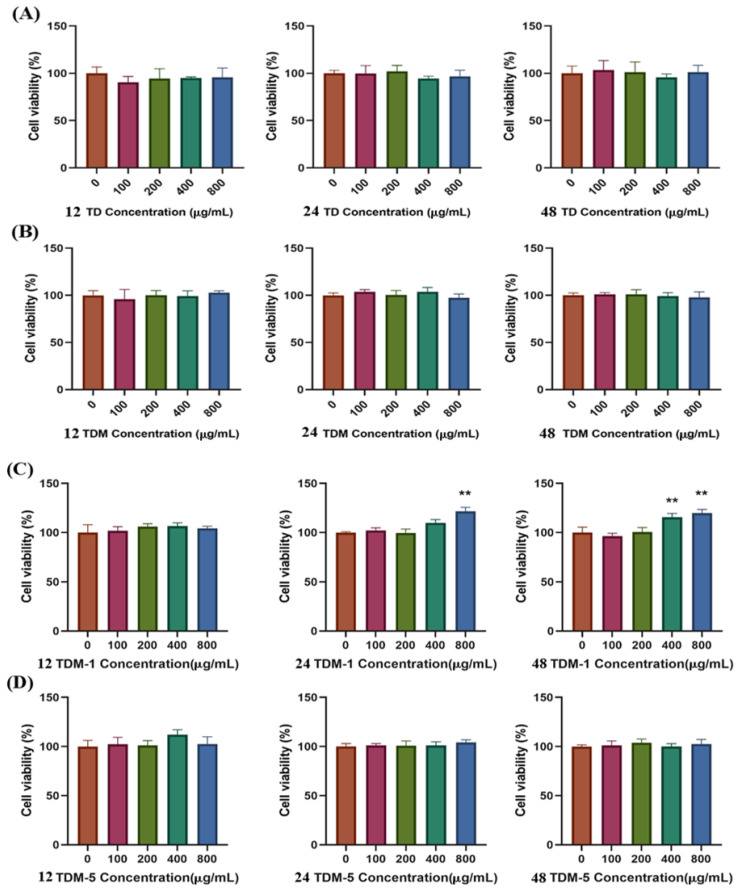
Cytotoxic Effects of TD, TDM, TDM-5, and TDM-1 on HaCaT Cells. (**A**) TD; (**B**) TDM; (**C**) TDM-1; (**D**) TDM-5; ** *p* < 0.01.

Before assessing photoprotective effects, the potential cytotoxicity of TD, TDM, TDM-5 and TDM-1 toward HaCaT keratinocytes was examined using the CCK-8 assay. Cells were treated with 100, 200, 400 and 800 μg·mL^−1^ of each sample for 12, 24 and 48 h (Figure 5). Across all concentrations and time points, no significant reduction in cell viability was observed compared with the untreated control. In some conditions, a slight increase in viability was detected, suggesting mild trophic effects.

These results indicate that TD, TDM, TDM-5 and TDM-1 are essentially non-toxic to HaCaT cells up to 800 μg·mL^−1^. For subsequent UVB-induced photoaging experiments, a working range of 0–400 μg·mL^−1^ was selected to ensure safety and allow observation of dose–response relationships.

#### 2.3.2. Establishment of a UVB-Induced Photoaging HaCaT Cell Model

**Figure 6:**
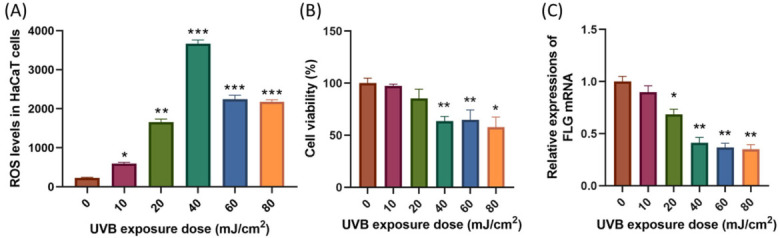
Establishment of a UVB-Induced Photoaging Model in HaCaT Cells. (**A**) Intracellular ROS levels in HaCaT cells after 12 h of recovery following 40 mJ·cm^−2^ UVB exposure; (**B**) Cell viability of HaCaT cells after 12-h irradiation with different doses of UVB; (**C**) FLG gene expression in HaCaT cells after 12 h of exposure to 40 mJ·cm^−2^ UVB. Data are presented as mean ± SD (n = 3). * *p* < 0.05, ** *p* < 0.01, *** *p* < 0.001 versus the control group.

To establish a reliable UVB-induced photoaging model, HaCaT cells were exposed to UVB at doses of 10, 20, 40, 60 and 80 mJ·cm^−2^, followed by 12 h of incubation. Intracellular ROS, cell viability and filaggrin (FLG) mRNA expression were quantified (Figure 6).

With increasing UVB dose, ROS levels rose steadily, while cell viability decreased in a dose-dependent manner. Compared with the control group, doses ≥ 40 mJ·cm^−2^ significantly reduced cell viability (*p* < 0.01), and at 60 and 80 mJ·cm^−2^ viability was further impaired, indicating partial irreversible damage. FLG mRNA, a key marker of epidermal barrier integrity, showed progressive downregulation with dose. At 40 mJ·cm^−2^, ROS levels reached a markedly elevated state (*p* < 0.001), while FLG expression substantially decreased (*p* < 0.01), yet overall viability remained adequate for subsequent intervention.

These findings indicate that 40 mJ·cm^−2^ UVB followed by 12 h incubation produces a stable oxidative stress condition with evident barrier impairment and moderate but not lethal injury. This dose was therefore chosen for constructing the UVB-induced photoaging model in subsequent experiments.

#### 2.3.3. Effects on UVB-Damaged HaCaT Cell Viability and Proliferation

**Figure 7:**
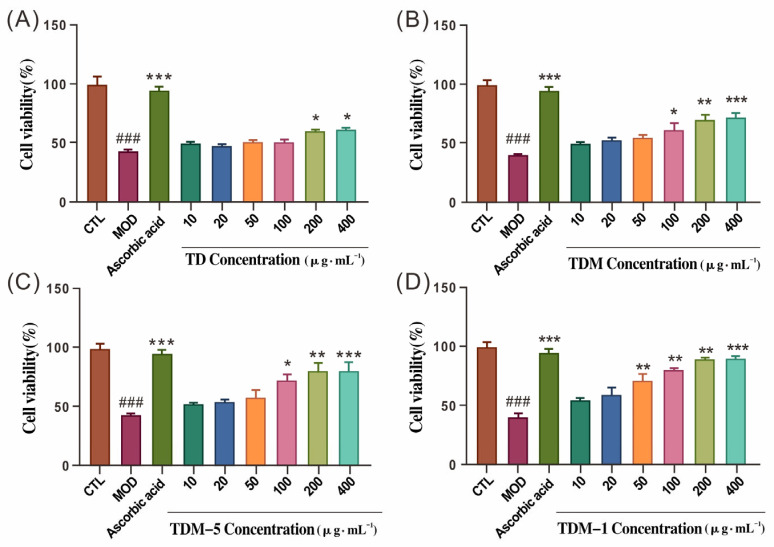
Effects of TD, TDM, TDM-5, and TDM-1 on the Viability of HaCaT Cells After UVB Irradiation. (**A**) Effects of TD components on cell viability; (**B**) Effects of TDM components on cell viability; (**C**) Effects of TDM-5 components on cell viability; (**D**) Effects of TDM-1 components on cell viability. Ascorbic acid: 5 μg·mL^−1^. ### *p* < 0.001, MOD vs. CTL; *** *p* < 0.001, ** *p* < 0.01, * *p* < 0.05, treatment groups vs. MOD.

After exposure to 40 mJ·cm^−2^ UVB, cell viability in the model group dropped to approximately 60% of that in the control group, confirming successful model establishment. Cells were then treated with TD, TDM, TDM-5 or TDM-1 at 10–400 μg·mL^−1^ for 12 h. As shown in Figure 7, all four samples improved cell viability compared with the UVB model group in a concentration-dependent manner.

TD began to exert a noticeable protective effect at 200 μg·mL^−1^. TDM and TDM-5 effectively restored viability at 100, 200 and 400 μg·mL^−1^, whereas TDM-1 showed significant protection at 200 and 400 μg·mL^−1^. At the highest concentration, cell viability in the TDM-1 group approached or exceeded that of the ascorbic acid group (5 μg·mL^−1^). These findings suggest that enzymatic hydrolysis and ultrafiltration treatment may influence the cytoprotective properties of *D. officinale* protein preparations against UVB-induced damage.

**Figure 8:**
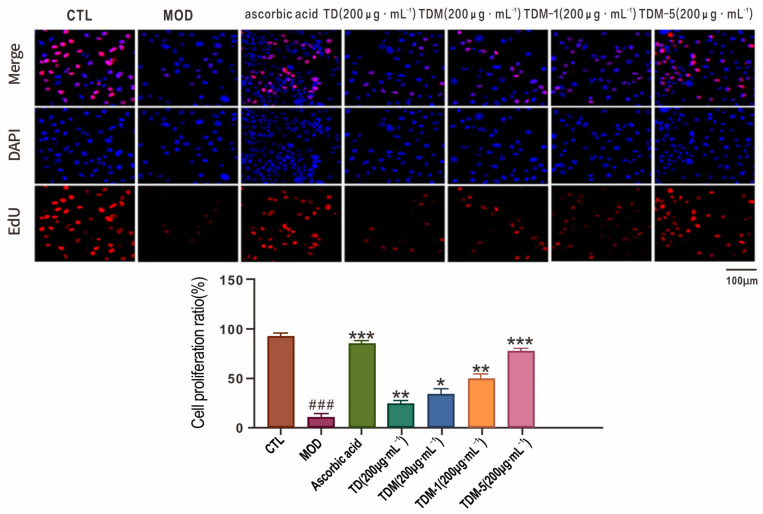
Proliferation of HaCaT Cells After UVB Irradiation Treated with TD, TDM, TDM-5, and TDM-1. Images were captured at 100× magnification. The concentrations of TD, TDM, TDM-5, and TDM-1 were 200 μg·mL^−1^, and Ascorbic acid was used as a positive control at 5 μg·mL^−1^. ### *p* < 0.001, MOD vs. CTL; *** *p* < 0.001, ** *p* < 0.01, * *p* < 0.05, treatment groups vs. MOD.

Cell proliferation was further assessed by EdU incorporation and DAPI nuclear staining (Figure 8). UVB irradiation markedly reduced the proportion of EdU-positive cells compared with the control group (### *p* < 0.001), indicating impaired DNA synthesis and proliferative activity. Ascorbic acid restored proliferation to near-normal levels (*** *p* < 0.001).

At 200 μg·mL^−1^, both TD and TDM increased EdU incorporation relative to the model group, with TDM showing a relatively stronger proliferative effect. TDM-5 and TDM-1 also noticeably increased the proportion of EdU-positive cells (*p* < 0.01 or *p* < 0.001). In some microscopic fields, the density of proliferating cells was comparable to that observed in the ascorbic acid group. These findings indicate that different ultrafiltration fractions may contribute to the recovery of proliferative activity in UVB-damaged keratinocytes.

### 2.4. Regulation of Cellular Antioxidant Systems by Hydrolysate Fractions

#### 2.4.1. Modulation of Intracellular ROS

**Figure 9:**
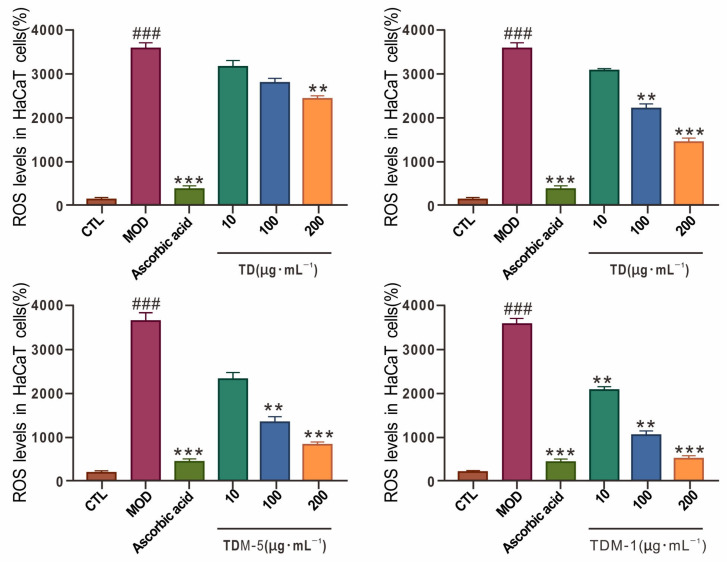
Effects of TD, TDM, TDM-5, and TDM-1 on Intracellular ROS Levels in HaCaT Cells After UVB Irradiation. Ascorbic acid: 5 μg·mL^−1^. ### *p* < 0.001, MOD vs. CTL; *** *p* < 0.001, ** *p* < 0.01, treatment groups vs. MOD.

UVB irradiation markedly elevates intracellular ROS in HaCaT cells, driving oxidative stress and subsequent damage. In the present study, the model group showed a marked increase in ROS compared with the control (*p* < 0.001; Figure 9). Ascorbic acid markedly suppressed ROS accumulation (*p* < 0.001), validating the assay.

Among the test samples, TD and TDM reduced ROS in a concentration-dependent manner, with TDM at 200 μg·mL^−1^ exhibiting a stronger inhibition than TD (*p* < 0.001), indicating that hydrolysis improves the ability of the protein to counteract UVB-induced oxidative stress. TDM-5 and TDM-1 further decreased ROS levels at 100 and 200 μg·mL^−1^, and their effects were more pronounced than those of TDM at the same concentrations (*p* < 0.01 or *p* < 0.001). These results suggest that the ultrafiltration fractions, particularly TDM-1, are more efficient at attenuating intracellular ROS in UVB-damaged keratinocytes, which aligns with their relatively stronger performance in DPPH and ·OH scavenging.

#### 2.4.2. Effects on MDA, SOD and T-AOC

**Figure 10:**
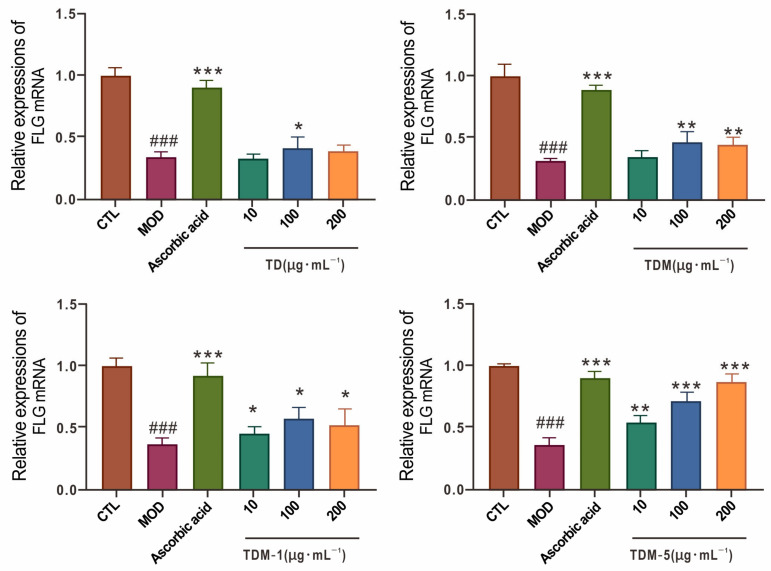
Effects of TD, TDM, TDM-5, and TDM-1 on MDA, SOD, and T-AOC Levels in HaCaT Cells After UVB Irradiation. Ascorbic acid: 5 μg·mL^−1^. ### *p* < 0.001, MOD vs. CTL; *** *p* < 0.001, ** *p* < 0.01, * *p* < 0.05, treatment groups vs. MOD.

To better characterize the antioxidant defense status, malondialdehyde (MDA), superoxide dismutase (SOD) and total antioxidant capacity (T-AOC) were measured (detailed numerical data are provided in Table S7). UVB irradiation effectively increased MDA content and decreased SOD activity and T-AOC compared with the control, indicating enhanced lipid peroxidation and compromised endogenous antioxidant capacity. Ascorbic acid reversed these changes and served as a reference for maximal correction (Figure 10).

Among the *D. officinale* fractions, TDM-5 at 200 μg·mL^−1^ and TDM-1 at 50–200 μg·mL^−1^ effectively reduced MDA (*p* < 0.001) and increased SOD activity and T-AOC (*p* < 0.01 or *p* < 0.001). Notably, TDM-1 at 100–200 μg·mL^−1^ improved SOD and T-AOC to levels comparable to those of 5 μg·mL^−1^ ascorbic acid, while simultaneously restoring MDA close to basal values. TD and TDM showed moderate or inconsistent improvements, and several doses did not reach statistical significance.

These findings indicate that TDM-5 and TDM-1 effectively re-establish the balance between oxidants and antioxidants in UVB-injured HaCaT cells, reducing membrane lipid peroxidation and enhancing enzymatic and non-enzymatic defense. TDM-1 showed a slightly relatively stronger overall regulatory effect, suggesting a central role in restoring the cellular antioxidant system.

### 2.5. Molecular Mechanisms Underlying the Anti-Photoaging Effects of Hydrolysate Fractions

#### 2.5.1. Effects on Epidermal Barrier and Hydration-Related Gene Expression

**Figure 11:**
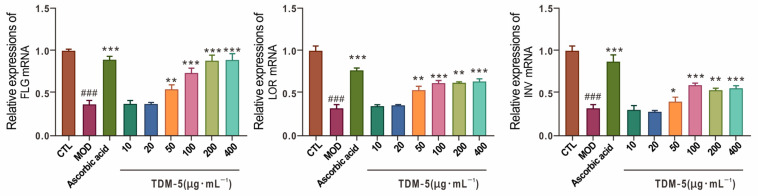
Modulation of epidermal barrier gene transcription by TDM-5 in HaCaT cells after UVB exposure. Ascorbic acid: 5 μg·mL^−1^. ### *p* < 0.001, MOD vs. CTL; *** *p* < 0.001, ** *p* < 0.01, * *p* < 0.05, Treatm0.01, treatment groups vs. MOD.

UVB exposure impairs epidermal barrier function and alters keratinocyte differentiation, reflected by downregulation of structural proteins such as filaggrin (FLG), involucrin (INV) and loricrin (LOR). In the present model, UVB markedly reduced FLG, INV and LOR mRNA levels compared with the control (*p* < 0.001; Figure 11), indicating barrier disruption and impaired terminal differentiation.

Ascorbic acid restored FLG expression nearly to control levels. Among the *D. officinale* fractions, TDM-5 at 50–200 μg·mL^−1^ effectively upregulated FLG mRNA, with the strongest effect at 200 μg·mL^−1^, where the expression approached that of ascorbic acid (*p* < 0.001). TDM-5 also markedly improved INV and LOR expression at 100–400 μg·mL^−1^ (*p* < 0.001), suggesting a pronounced ability to promote keratinocyte differentiation and barrier repair. TDM-1 enhanced FLG to a lesser extent at higher concentrations, while TD had no significant effect on FLG.

**Figure 12:**
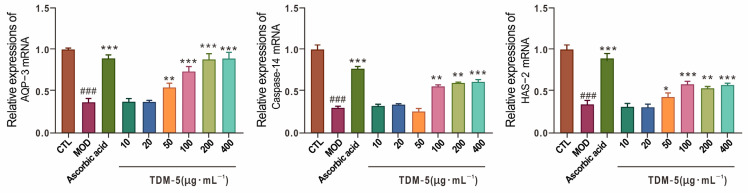
TDM-5 enhances the expression of hydration-associated genes in UVB-treated HaCaT cells. Ascorbic acid: 5 μg·mL^−1^. ### *p* < 0.001, MOD vs. CTL; *** *p* < 0.001, ** *p* < 0.01, * *p* < 0.05, treatment groups vs. MOD.

Hydration of the stratum corneum depends on aquaporin-3 (AQP-3), Caspase-14 and hyaluronan synthase-2 (HAS-2). AQP-3 mediates water and glycerol transport, Caspase-14 participates in FLG processing into natural moisturizing factor components, and HAS-2 is essential for hyaluronan synthesis. UVB noticeably downregulated AQP-3, Caspase-14 and HAS-2 mRNA levels (Figure 12), reflecting impaired hydration and extracellular matrix support.

TDM-5 at 100–400 μg·mL^−1^ markedly increased AQP-3, Caspase-14 and HAS-2 expression (*p* < 0.01 or *p* < 0.001), with a clear dose–response trend. Considering the improvement in FLG, INV and LOR, these data suggest that TDM-5 not only counteracts oxidative injury but also reinforces both the structural and hydrating components of the epidermal barrier. This multifaceted effect is likely to contribute to the prevention of UVB-induced dryness, barrier dysfunction and subsequent sensitivity.

#### 2.5.2. Modulation of Pro-Inflammatory Cytokines and Matrix Metalloproteinases

**Figure 13:**
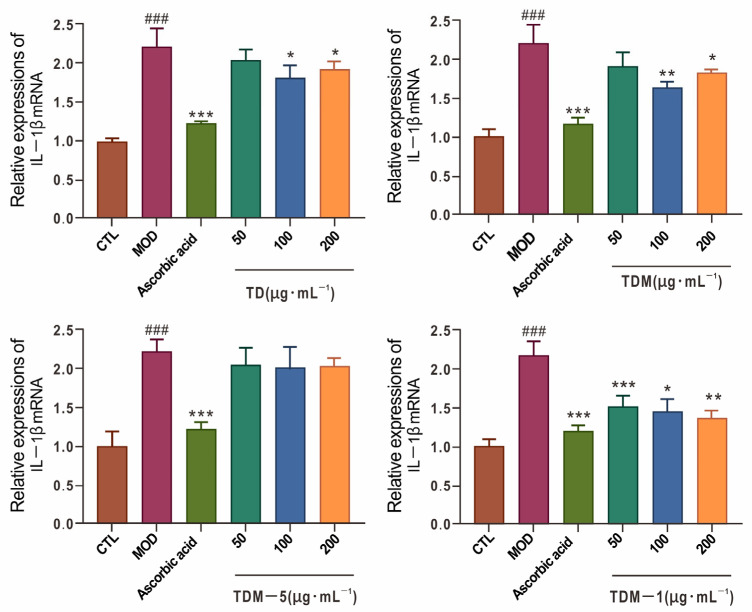
Effects of TD, TDM, TDM-5, and TDM-1 on IL-1β mRNA Expression in HaCaT Cells After UVB Irradiation. Ascorbic acid: 5 μg·mL^−1^. ### *p* < 0.001, MOD vs. CTL; *** *p* < 0.001, ** *p* < 0.01, * *p* < 0.05, treatment groups vs. MOD.

UVB-induced ROS can activate inflammatory pathways and increase the production of cytokines such as IL-1β, IL-6, IL-8 and TNF-α, as well as matrix metalloproteinases (MMPs) that degrade dermal collagen. In this study, UVB exposure markedly elevated IL-1β mRNA in HaCaT cells (*p* < 0.001; Figure 13), indicating a strong inflammatory response. Ascorbic acid significantly lowered IL-1β expression toward basal levels.

After treatment with *D. officinale* fractions, TD at 100–200 μg·mL^−1^, TDM at 100 μg·mL^−1^ and TDM-1 at 200 μg·mL^−1^ markedly reduced IL-1β mRNA (*p* < 0.05 or *p* < 0.01), while TDM-5 showed no significant effect within the tested range. This pattern supports a functional distinction, with TDM-1 being more involved in anti-inflammatory regulation and TDM-5 being more specialized in barrier repair.

**Figure 14:**
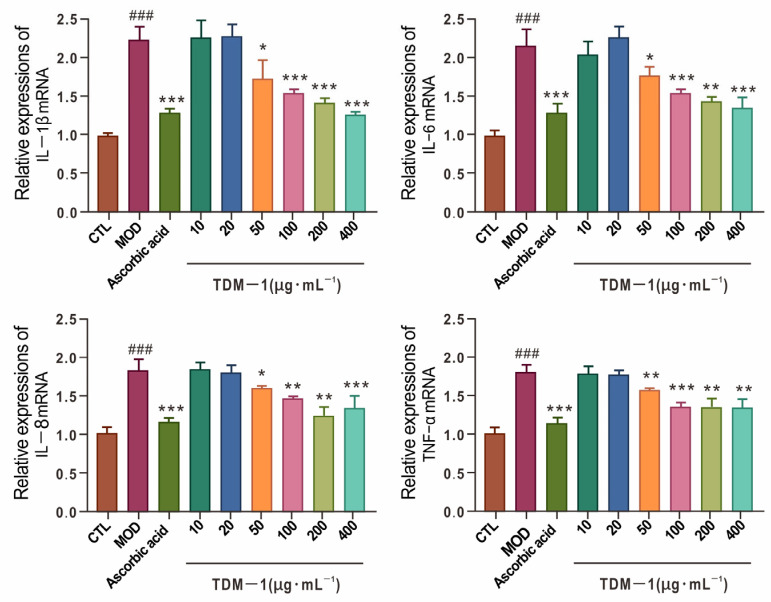
TDM-1 modulates inflammatory gene expression in HaCaT cells following UVB exposure. The concentration of ascorbic acid was 5 μg·mL^−1^. ### *p* < 0.001, MOD vs. CTL; *** *p* < 0.001, ** *p* < 0.01, * *p* < 0.05, experimental groups vs. MOD.

To obtain a broader view of the inflammatory profile, IL-1β, IL-6, IL-8 and TNF-α mRNA levels were further measured after TDM-1 treatment (Figure 14). UVB substantially upregulated all four cytokines (*p* < 0.001). TDM-1 at 100 μg·mL^−1^ decreased IL-1β, IL-8 and TNF-α (*p* < 0.01 or *p* < 0.001), and at 200 μg·mL^−1^ significantly reduced IL-1β, IL-6 and IL-8 mRNA levels (*p* < 0.001). These results indicate that TDM-1 exerts a broad anti-inflammatory effect in UVB-damaged keratinocytes, likely mediated through upstream signal modulation.

**Figure 15:**
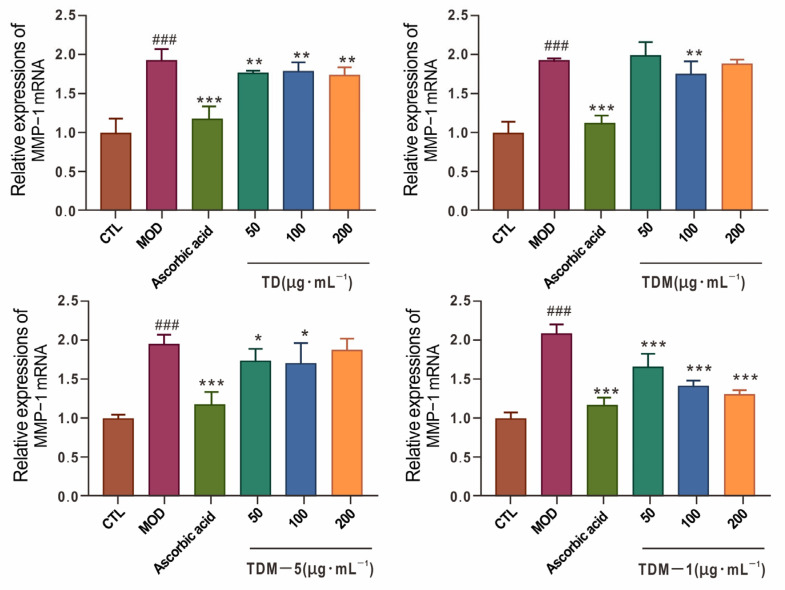
Effects of TD, TDM, TDM-5, and TDM-1 on MMP-1 mRNA Expression in HaCaT Cells After UVB Irradiation. Ascorbic acid: 5 μg·mL^−1^. ### *p* < 0.001, MOD vs. CTL; *** *p* < 0.001, ** *p* < 0.01, * *p* < 0.05, treatment groups vs. MOD.

**Figure 16:**
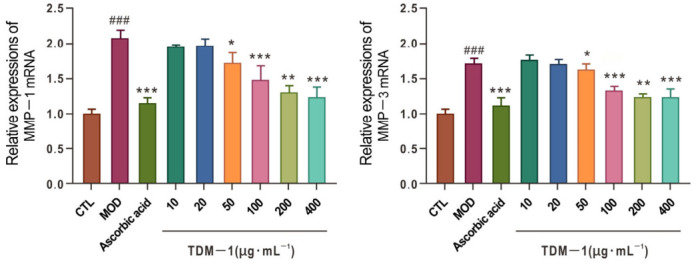
Effects of TDM-1 on the mRNA Expression of Matrix Metalloproteinases in HaCaT Cells After UVB Irradiation. Ascorbic acid: 5 μg·mL^−1^. ### *p* < 0.001, MOD vs. CTL; *** *p* < 0.001, ** *p* < 0.01, * *p* < 0.05, treatment groups vs. MOD.

MMP-1 and MMP-3 are key enzymes responsible for degradation of type I and III collagen. In the model, UVB markedly increased MMP-1 mRNA (*p* < 0.001; Figure 15), and both MMP-1 and MMP-3 were elevated in a separate experiment (Figure 16). TDM-1 downregulated MMP-1 and MMP-3 expression in a dose-dependent manner, with significant inhibition already evident at 100 μg·mL^−1^ and further enhanced at 200 μg·mL^−1^ (*p* < 0.001). TD and TDM also decreased MMP-1 in the 50–200 μg·mL^−1^ range, while TDM-5 showed a weaker effect, most apparent at 200 μg·mL^−1^.

These data collectively indicate that TDM-1 plays a predominant role in suppressing UVB-induced inflammatory responses and extracellular matrix degradation, whereas TDM-5 mainly contributes to barrier and hydration recovery. The two fractions therefore act in a complementary fashion to counteract photoaging.

#### 2.5.3. Regulation of MAPK/AP-1 Signaling

**Figure 17:**
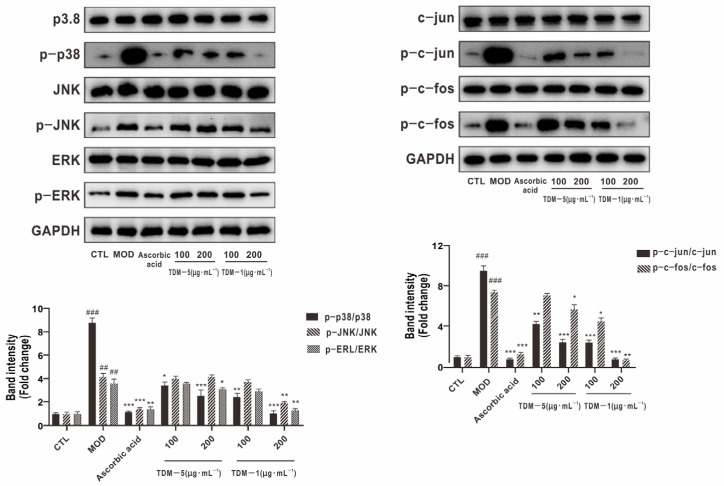
Effects of TDM-5 and TDM-1 Components on the Expression of MAPK/AP-1 Signaling Pathway-Related Proteins in Photoaged HaCaT Cells. The concentration of ascorbic acid was 5 μg·mL^−1^. ### *p* < 0.001, ## *p* < 0.01, MOD vs. CTL; *** *p* < 0.001, ** *p* < 0.01, * *p* < 0.05, experimental groups vs. MOD.

The MAPK family (p38, JNK and ERK1/2) and their downstream AP-1 transcription factors (c-Jun/c-Fos) are central mediators of UVB-induced oxidative and inflammatory signaling. Western blot analysis showed that total p38, JNK and ERK1/2 levels remained relatively unchanged across groups, while their phosphorylated forms (p-p38, p-JNK and p-ERK1/2) increased markedly in the UVB model group (Figure 17), indicating activation of MAPK pathways.

Treatment with TDM-5 or TDM-1 decreased p-p38 and the p-p38/p38 ratio, suggesting inhibition of p38-mediated inflammatory signaling. Both fractions also reduced p-JNK and the p-JNK/JNK ratio, indicating attenuation of stress-related JNK activation. TDM-1 at 200 μg·mL^−1^ additionally lowered p-ERK1/2 levels, suggesting that ERK1/2 signaling can be modulated at higher doses.

In parallel, phosphorylation of c-Jun and c-Fos, as well as the p-c-Jun/c-Jun and p-c-Fos/c-Fos ratios, were significantly increased after UVB exposure and were brought down by TDM-5 and TDM-1. This indicates that both fractions limit AP-1 activation, which is in agreement with the observed reduction in MMP-1/MMP-3 expression. Since AP-1 is a key driver of collagen-degrading MMPs, modulation of MAPK/AP-1 signaling by these fractions may be associated with ECM preservation and attenuation of wrinkle formation associated with photoaging.

#### 2.5.4. Regulation of NF-κB Signaling

**Figure 18:**
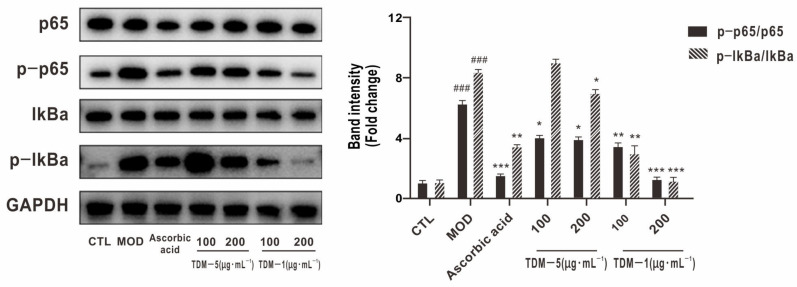
Effects of TDM-5 and TDM-1 Components on the Expression of NF-κB Signaling Pathway-Related Proteins in Photoaged HaCaT Cells. The concentration of ascorbic acid was 5 μg·mL^−1^. ### *p* < 0.001, MOD vs. CTL; *** *p* < 0.001, ** *p* < 0.01, * *p* < 0.05, experimental groups vs. MOD.

NF-κB is another critical transcription factor involved in inflammation and ECM remodeling. In resting cells, NF-κB is retained in the cytoplasm by binding to IκBα. Upon stimulation, IκBα is phosphorylated, ubiquitinated and degraded, allowing NF-κB to translocate into the nucleus and activate target genes. In the present study, UVB significantly increased p-IκBα, the p-IκBα/IκBα ratio and the p-p65/NF-κB ratio (Figure 18), indicating activation of the NF-κB pathway.

TDM-5 and TDM-1 both reduced p-IκBα and p-p65/NF-κB levels and partially restored IκBα protein. This observation implies that the fractions may modulate NF-κB signaling by influencing IκBα phosphorylation and degradation, thus limiting NF-κB nuclear translocation and transcriptional activity. Given that NF-κB regulates IL-1β, IL-6, IL-8, TNF-α and several MMPs, the observed inhibition of these downstream targets by TDM-1 is consistent with NF-κB modulation. Combined with the MAPK/AP-1 data, these results point to coordinated suppression of multiple pro-inflammatory and pro-degradative pathways.

#### 2.5.5. Regulation of TGF-β/Smad Signaling

**Figure 19:**
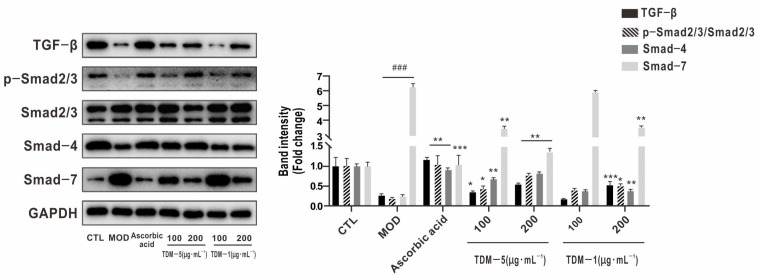
Effects of TDM-5 and TDM-1 Components on the Expression of TGF-β Signaling Pathway-Related Proteins in Photoaged HaCaT Cells. The concentration of ascorbic acid was 5 μg·mL^−1^. ### *p* < 0.001, MOD vs. CTL; *** *p* < 0.001, ** *p* < 0.01, * *p* < 0.05, experimental groups vs. MOD.

The TGF-β/Smad pathway plays a crucial role in maintaining ECM homeostasis, associated with collagen-related protein expression and modulating inflammation. UVB exposure substantially reduced TGF-β, Smad2/3, p-Smad2/3 and Smad4 protein levels, while increasing the inhibitory Smad7 (*p* < 0.001; Figure 19), indicating suppression of TGF-β signaling and a shift toward ECM degradation rather than synthesis.

After treatment with TDM-5 or TDM-1, TGF-β, Smad2/3, p-Smad2/3 and Smad4 levels increased compared with the model group, while Smad7 decreased slightly. These changes suggest partial restoration of TGF-β/Smad signaling, likely associated with reduced competitive inhibition by Smad7 and enhanced formation of active Smad2/3–Smad4 complexes. These changes were associated with improved ECM-related signaling and potential collagen maintenance, counterbalancing the pro-degradative activity driven by MAPK/AP-1 and NF-κB.

#### 2.5.6. Integrated Mechanistic Interpretation

The present findings indicate that *D. officinale* protein hydrolysates and their ultrafiltration fractions protect HaCaT keratinocytes from UVB-induced photoaging through multi-level and complementary mechanisms. At the physicochemical level, enzymatic hydrolysis and size fractionation generate peptide mixtures with distinct antioxidant profiles. At the cellular level, these fractions limit ROS accumulation, restore endogenous antioxidant defenses, promote barrier and hydration-related gene expression, and suppress inflammatory cytokines and MMPs.

**Figure 20:**
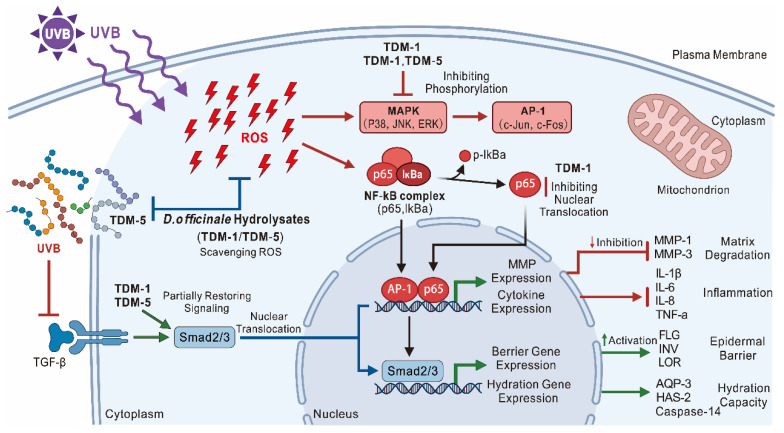
Mechanism diagram.

Mechanistically, TDM-5 is more closely associated with barrier repair and hydration, reflected by robust upregulation of FLG, INV, LOR, AQP-3, Caspase-14 and HAS-2, whereas TDM-1 exerts stronger anti-inflammatory and anti-MMP effects associated with marked inhibition of NF-κB and MAPK/AP-1 signaling and downregulation of IL-1β, IL-6, IL-8, TNF-α, MMP-1 and MMP-3. Both fractions contribute to reactivation of TGF-β/Smad signaling, thereby supporting ECM maintenance and counteracting collagen loss (Figure 20).

These complementary actions suggest that *D. officinale*–derived peptides, particularly the 10–50 kDa fraction (TDM-5) and the <10 kDa fraction (TDM-1), represent promising multifunctional anti-photoaging candidates that combine direct antioxidant activity with targeted modulation of key signaling pathways regulating skin barrier integrity, inflammation and matrix remodeling.

#### 2.5.7. Limitation and Future Perspective Section

Several limitations should also be acknowledged. First, the present study was based on an in vitro HaCaT cell model and therefore cannot fully reflect the complexity of skin tissue under physiological conditions. Second, it should be noted that TDM-1 and TDM-5 are ultrafiltration fractions rather than purified peptides, and their bioactivities may be influenced not only by molecular weight distribution but also by peptide sequence, amino acid composition, hydrophobicity, charge properties, and potential co-existing non-peptide components. Therefore, the observed differences in biological activities should be interpreted cautiously. Further studies involving advanced compositional characterization techniques, such as LC-MS/MS and amino acid profiling, are needed to better elucidate the structure–activity relationships of these hydrolysate fractions. Although alterations in MAPK/AP-1, NF-κB and TGF-β/Smad pathway-related proteins were observed after treatment, the present study only suggests an association between pathway modulation and the observed photoprotective effects. Additional mechanistic studies using pathway inhibitors, activators or gene-silencing approaches are required to further verify the causal involvement of these signaling pathways.

Overall, the present study primarily focused on evaluating the photoprotective activity of DOPH peptide fractions rather than identifying individual peptide sequences. Although peptide sequencing analysis could provide further insights into structure–activity relationships, such analysis was beyond the scope of the current study and will be considered in future investigations. The present findings expand the functional value of *D. officinale* beyond its commonly studied polysaccharides and support the potential use of its protein-derived hydrolysates as multifunctional ingredients for food, nutraceutical, or cosmeceutical applications.

## 3. Materials and Methods

### 3.1. Materials

#### 3.1.1. Plant Material

Dried stems of *Dendrobium officinale* Kimura et Migo were purchased from Guangzhou Anjiehui Trading Co., Ltd. (Guangzhou, China) and authenticated as *D. officinale* (Orchidaceous). The material was ground to a fine powder, passed through a 60-mesh sieve, and stored in sealed containers at room temperature until its use.

#### 3.1.2. Chemicals and Reagents

Sodium hydroxide (NaOH), hydrochloric acid (HCl), sulfuric acid (H_2_SO_4_), copper sulfate (CuSO_4_·5H_2_O), potassium sulfate (K_2_SO_4_), boric acid (H_3_BO_3_), and absolute ethanol were of analytical grade (Sinopharm Chemical Reagent Co., Ltd., Shanghai, China). Ascorbic acid (vitamin C, purity ≥ 99%, Shanghai Yuanye Bio-Technology Co., Ltd., Shanghai, China)

Neutral protease and papain were obtained from Solarbio Life Science (Beijing, China). BCA protein assay kits, CCK-8 cell viability kits, ROS, SOD, MDA and T-AOC detection kits, BCA protein assay kits, SDS-PAGE gel preparation kits, silver staining kits, RIPA lysis buffer, TBST buffer and ECL chemiluminescent substrates were purchased from Beyotime Biotechnology (Shanghai, China) or Solarbio (Beijing, China).

DPPH, ABTS, and potassium persulfate used in the antioxidant assays were purchased from Aladdin (Shanghai, China) or Macklin Biochemical Co., Ltd. (Shanghai, China). Other reagents for FRAP and hydroxyl radical scavenging assays, including ferric chloride, ferrous sulfate, potassium ferricyanide, salicylic acid, and trichloroacetic acid, were of analytical grade from domestic suppliers.

Dulbecco’s modified Eagle’s medium (DMEM), fetal bovine serum (FBS), penicillin–streptomycin, and trypsin–EDTA were obtained from Gibco (Thermo Fisher Scientific, Waltham, MA, USA). HaCaT human keratinocytes were purchased from FuHeng Biology (Shanghai, China). EdU-488 proliferation assay kits, CCK-8 kits, and ROS detection kits were supplied by Beyotime (Shanghai, China).

TRIzol Reagent, reverse transcription kits (HyperScript™ First-Strand cDNA Synthesis SuperMix), and SYBR Green qPCR Master Mix (HotStart™ 2×) were obtained from APExBIO (Houston, TX, USA) or other commercial suppliers as specified in the text. Primary antibodies against p38 MAPK, JNK, ERK1/2, p-p38, p-JNK, p-ERK1/2, c-Jun, c-Fos, p-c-Jun, p-c-Fos, NF-κB p65, IκBα, p-IκBα, TGF-β, Smad2/3, p-Smad2/3, Smad4, Smad7, and GAPDH were purchased from Thermo Fisher Scientific (Waltham, MA, USA) and Proteintech (Wuhan, China).

Primers for human FLG, AQP3, Caspase-14, HAS2, IL1B, IL6, IL8, TNFA, MMP1, MMP3, INV, and LOR were synthesized by Sangon Biotech (Shanghai, China).

#### 3.1.3. Instruments

The main instruments used in this study included an analytical balance and microbalance (Ohaus, Changzhou, China), pH meter (ST2100, Ohaus, Changzhou, China), thermostatic water bath (DK-S26, Shanghai Jinghong Laboratory Equipment Co., Ltd., Shanghai, China), ultrasonic cleaner (KQ-600E, Jielimei, Kunshan, China), rotary evaporator (RE-52, Yarong, Shanghai, China), refrigerated centrifuge (S418R, Eppendorf, Hamburg, Germany), vacuum freeze dryer (FD-2B, Bilang Instrument Manufacturing Co., Ltd., Shanghai, China), and a UVB 311 nm lamp (TL20W, Philips, Amsterdam, The Netherlands).

Cell culture and molecular analyses were performed using a CO_2_ incubator (Series 8000, Thermo Fisher Scientific, Waltham, MA, USA), biosafety cabinet (BCM-1000A, Suzhou Antai Air Technology Co., Ltd., Suzhou, China), inverted phase-contrast microscope (CKX53, Olympus, Tokyo, Japan), microplate reader (Multiskan MK3, Thermo Fisher Scientific), flow cytometer (BD Biosciences, San Jose, CA, USA), PCR thermocycler (A200, Hangzhou LongGene, Hangzhou, China), real-time PCR system (CFX96, Bio-Rad, Hercules, CA, USA), wet transfer system for Western blotting (Bio-Rad, Hercules, CA, USA), and chemiluminescence imaging system (Tanon-5200CE, Tanon, Shanghai, China).

### 3.2. Extraction of Dendrobium officinale Protein

Crude *Dendrobium officinale* protein (TD) was prepared using an alkaline extraction–acid precipitation procedure. Briefly, powdered *D. officinale* stems were dispersed in NaOH solution and extracted in a thermostatic water bath with continuous stirring. The extract was filtered to remove insoluble residues, and the filtrate was neutralized with HCl.

Proteins were then precipitated by adjusting the solution to their isoelectric point (pH 4.0), collected by centrifugation, re-dissolved in deionized water, and dialyzed (3 kDa cut-off) against deionized water to remove salts and low-molecular-weight impurities. The dialyzed solution was filtered (0.45 μm) and lyophilized to obtain TD, which was stored at −20 °C until use.

The extraction conditions (temperature, time, NaOH concentration, and solid–liquid ratio) were optimized using single-factor experiments and Box–Behnken response surface methodology. The final operational parameters and model fitting are provided in Supplementary Method S1 and Table S4.

### 3.3. Preparation of D. officinale Protein Hydrolysates and Ultrafiltration Fractions

The preparation of *D. officinale* protein hydrolysates was based on a previously described method [34], with minor modifications. Briefly, 0.15 g of *D. officinale* protein powder was dissolved in 50 mL deionized water to obtain a 0.3% (*w*/*v*) protein solution, and the pH was adjusted to 6.9 using 0.1 mol·L^−1^ HCl or NaOH.

Papain and neutral protease were mixed at a 1:1 activity ratio and added to the protein solution at a concentration of 5000 U per g protein. The mixture was incubated under controlled conditions for enzymatic hydrolysis. At the end of the reaction, the enzyme was inactivated in a 90 °C water bath for 15 min, and the solution was cooled to room temperature and centrifuged at 5000 rpm for 15 min. The supernatant was collected as a crude hydrolysate.

The crude hydrolysate was fractionated as follows: filtration through a 0.45 μm membrane to obtain the total hydrolysate (TDM). Ultrafiltration through 50 kDa and 10 kDa membranes to obtain the 10–50 kDa fraction (TDM-5); ultrafiltration through a 10 kDa membrane to obtain the <10 kDa fraction (TDM-1).

All filtrates were freeze-dried and stored at −20 °C until use. Detailed filtration and drying conditions are provided in Supplementary Methods S2.

### 3.4. Determination and Optimization of the Degree of Hydrolysis

The degree of hydrolysis (DH) of *D. officinale* proteins was determined using the OPA method. A freshly prepared OPA working solution was used to quantify the free amino groups, with serine as the standard. Samples were diluted to appropriate concentrations, incubated with OPA reagent for 2 min at room temperature, and the absorbance was measured at 340 nm. DH was calculated from the difference in free amino groups before and after hydrolysis, using a total peptide bond value (h_tot) of 8 for *D. officinale* protein. The detailed preparation of the OPA reagent, standard curve construction, and DH formula are described in Supplementary Methods S3.

Single-factor experiments were first conducted to examine the effects of hydrolysis time (1–5 h), pH (5.0–9.0), temperature (45–65 °C), and enzyme dosage (3000–11,000 U·g^−1^ protein) on DH. In all experiments, the substrate concentration was 0.3% (*w*/*v*), and other factors were kept constant when one variable was altered.

Based on the single-factor results, a Box–Behnken design was applied to optimize three key parameters: hydrolysis time (A, 2–4 h), enzyme dosage (B, 5000–9000 U·g^−1^), and pH (C, 6–8). DH was used as the response variable. The experimental data were fitted to a second-order polynomial model, and ANOVA was performed using Design-Expert 13 to evaluate the model significance and obtain the optimum hydrolysis conditions. The design matrix and regression analysis are presented in Supplementary Table S5.

### 3.5. SDS–PAGE Analysis of Molecular Weight Distribution

The molecular weight distributions of *D. officinale* protein (TD), the crude hydrolysate (TDM), and the ultrafiltration fractions (TDM-5 and TDM-1) were analyzed by SDS–PAGE with 5% stacking and 10% resolving gels. Samples were dissolved in deionized water, mixed with loading buffer, and loaded alongside a molecular weight marker. Electrophoresis was performed under constant voltage, and the gels were silver-stained to visualize the protein bands.

Detailed compositions of gels, buffers, and the silver staining protocol are provided in Supplementary Methods S4.

### 3.6. In Vitro Antioxidant Assays

The antioxidant activities of TD, TDM, TDM-5, and TDM-1 were evaluated using four complementary assays: DPPH radical scavenging, ABTS radical scavenging, ferric-reducing antioxidant power (FRAP), and hydroxyl radical(·OH) scavenging.

For the DPPH assay, the samples were mixed with freshly prepared DPPH ethanolic solution and incubated for 30 min at room temperature in the dark. Absorbance was measured at 517 nm, and DPPH scavenging activity was calculated relative to the appropriate sample and reagent blanks.

ABTS radical scavenging was determined using an ABTS^+^· working solution adjusted to an absorbance of 0.70 ± 0.02 at 734 nm wavelength. Sample solutions were mixed with the ABTS working solution, incubated for 10 min in the dark, and the decrease in absorbance at 734 nm was recorded.

For FRAP, the reducing capacity of the samples was measured by the reduction of Fe^3+^ to Fe^2+^ in the presence of potassium ferricyanide and TCA, followed by complex formation with FeCl_3_. Absorbance was measured at 700 nm and converted to FRAP values using a Fe^2+^ standard curve.

Hydroxyl radical scavenging activity was assessed using the salicylic acid method, in which hydroxyl radicals are generated by the Fenton reaction (Fe^2+^/H_2_O_2_) and react with salicylic acid. The presence of antioxidants reduces the formation of chromogenic products, which were monitored at 517 nm.

The exact reagent compositions, calculation formulas, and assay conditions are provided in Supplementary Methods S5.

### 3.7. Cell Culture, Treatments and UVB-Induced Photoaging Model

HaCaT keratinocytes were maintained in DMEM supplemented with 10% FBS and 1% penicillin–streptomycin at 37 °C in a humidified incubator with 5% CO_2_. Cells in the logarithmic growth phase were used for the experiments.

TD, TDM, TDM-5, and TDM-1 were dissolved in deionized water, sterilized by filtration (0.22 μm), and diluted with culture medium to the desired concentrations immediately before use. Ascorbic acid (vitamin C) was prepared as a sterile stock solution and used as positive control. The concentration of 5 μg·mL^−1^ was selected based on preliminary experiments and previous studies demonstrating its protective effect against UVB-induced oxidative damage in HaCaT cells without inducing cytotoxicity.

For cytotoxicity testing, HaCaT cells were seeded in 96-well plates and treated with 100–800 μg·mL^−1^ of each sample for 12, 24, or 48 h. Cell viability was assessed using the CCK-8 assay and expressed as a percentage of the untreated control.

To establish a UVB-induced photoaging model, HaCaT cells were seeded in 96-well or 6-well plates and allowed to attach. The medium was removed, and the cells were washed with PBS before UVB exposure. A UVB lamp (15 W, 280–380 nm, peak at 315 nm) was positioned approximately 10 cm above each plate. Cells were irradiated at doses of 0–80 mJ·cm^−2^ and incubated with fresh medium for 12 or 24 h. The UVB irradiation range (0–80 mJ·cm^−2^) was selected based on preliminary experiments and previous studies to establish a stable photoaging model with significant oxidative damage while maintaining sufficient cell viability for subsequent analyses. Viability, intracellular ROS, and FLG mRNA levels were measured to determine the optimal modeling dose, which was used in subsequent experiments. The experimental groups were defined as follows: the control group consisted of untreated HaCaT cells without UVB irradiation; the model group consisted of cells exposed to UVB irradiation without sample treatment; the positive control group consisted of UVB-irradiated cells treated with ascorbic acid; and the treatment groups consisted of UVB-irradiated cells treated with TD, TDM, TDM-5, or TDM-1 at the indicated concentrations.

For protection assays, after modeling at the selected UVB dose, cells were incubated with TD, TDM, TDM-5, TDM-1, or ascorbic acid at different concentrations for 12 h.

### 3.8. CCK-8 Assay and EdU Proliferation Assay

Cell viability in the UVB model and after treatment was quantified using the CCK-8 assay. After treatment, the cells were incubated with CCK-8 working solution (diluted 1:10 in culture medium) for 40 min, and the absorbance at 450 nm was measured. Cell viability was calculated relative to the control and model groups.

Cell proliferation after UVB irradiation and treatment was assessed using an EdU incorporation assay. HaCaT cells were seeded in 6-well plates, subjected to UVB irradiation and drug treatments as described, and incubated with EdU according to the manufacturer’s instructions. The cells were fixed, stained with EdU-specific fluorescent azide, and counterstained with DAPI. Images were acquired using a fluorescence microscope, and the proportion of EdU-positive cells was used as an index of proliferation.

### 3.9. Measurement of ROS, Antioxidant Indices and Gene Expression

Intracellular ROS levels were measured using flow cytometry with a commercial ROS probe. After UVB irradiation and treatment, cells were incubated with an ROS probe in serum-free medium at 37 °C in the dark, washed, and analyzed by flow cytometry. The mean fluorescence intensity was used to quantify ROS.

MDA content, SOD activity, and total antioxidant capacity (T-AOC) were determined using commercial assay kits according to the manufacturer’s instructions. HaCaT cells were seeded in 6-well plates, subjected to UVB irradiation and treatment, collected, and lysed. The supernatants were used for biochemical measurements.

For RT-qPCR, total RNA was extracted using TRIzol reagent after UVB irradiation and treatment. cDNA was synthesized from total RNA, and RT-qPCR was performed using an SYBR Green master mix. GAPDH was used as the housekeeping gene (internal control) for normalization of gene expression levels, and relative gene expression was calculated using the 2^−ΔΔCt^ method. The genes analyzed included *FLG*, *INV*, *LOR*, *AQP-3*, *Caspase-14*, HAS-2, *IL-1β*, *IL-6*, *IL-8*, *TNF-α*, *MMP-1* and *MMP-3*. The complete list of primer sequences is provided in Table S6.

### 3.10. Western Blot Analysis of Signaling Pathways

To investigate the molecular potential mechanisms involved, Western blotting was used to evaluate proteins in the MAPK (p38, JNK, ERK1/2), AP-1 (c-Jun, c-Fos), NF-κB (p65, IκBα) and TGF-β/Smad pathways. After UVB irradiation and treatment, HaCaT cells were lysed in RIPA or equivalent buffer containing protease and phosphatase inhibitors. Protein concentrations were measured; equal amounts of protein were separated by SDS–PAGE and transferred onto PVDF membranes.

Membranes were blocked and incubated with primary antibodies against total and phosphorylated forms of the target proteins at 4 °C overnight, followed by HRP-conjugated secondary antibodies. Protein bands were visualised using chemiluminescent substrate and quantified with ImageJ1.53. Relative protein levels were normalised to housekeeping proteins or to total protein for phosphorylation ratios. Detailed electrophoresis and transfer conditions are provided in Supplementary Methods S6.

### 3.11. Statistical Analysis

Data are expressed as mean ± standard deviation (SD) of at least three independent experiments. Data were analyzed by one-way ANOVA followed by Tukey’s multiple comparison test. Differences were considered statistically significant at *p* < 0.05.

## 4. Conclusions

In this work, *Dendrobium officinale* protein was hydrolysed using a mixed papain/neutral protease system, and the process was optimized by response surface methodology to obtain a high degree of hydrolysis and well-defined peptide fractions (TDM, TDM-5 and TDM-1). SDS–PAGE confirmed a clear shift toward lower-molecular-weight peptides after enzymatic treatment and ultrafiltration. In vitro, enzymatic hydrolysis enhanced the antioxidant properties of *D. officinale* protein, and certain ultrafiltration fractions, particularly TDM-1 and TDM-5, showed stronger DPPH, ABTS and ·OH scavenging activities than the native protein. In UVB-irradiated HaCaT cells, these fractions exhibited good safety, improved cell viability and proliferation, reduced ROS accumulation and MDA production, and increased SOD and T-AOC levels. TDM-5 and TDM-1 increased the expression of key barriers and hydration genes and reduced the expression of inflammatory cytokines and MMPs. Changes in MAPK/AP-1, NF-κB and TGF-β/Smad signaling were consistent with reduced oxidative stress, inflammation and matrix degradation. These findings suggest that *D. officinale* protein-derived peptides, especially TDM-5 (10–50 kDa) and TDM-1 (<10 kDa), have potential as multifunctional cosmetic or nutraceutical ingredients for protecting skin from UVB-induced oxidative damage and photoaging.

## Data Availability

The data presented in this study are available within the article and the Supplementary Materials. Additional raw data supporting the findings of this study are available from the corresponding author upon reasonable request.

## Supplementary Materials

The following supporting information can be downloaded at: https://www.mdpi.com/article/10.3390/molecules31121990/s1, Table S1: RT-PCR Filaggrin primer sequences. Table S2: IL-1β Gene Primer Sequences. Table S3: MMP-1 gene primer sequences. Table S4: Response surface methodology design and analysis for protein extraction. Table S5: Optimization of protein hydrolysis using response surface methodology. Table S6: Primer sequences used for RT-qPCR. Table S7: Effects of TD, TDM, TDM-5, and TDM-1 on MDA, SOD, and T-AOC Levels in HaCaT Cells After UVB Irradiation. Ascorbic acid: 5 μg·mL^−1^. ### *p* < 0.001, MOD vs. CTL; *** *p* < 0.001, ** *p* < 0.01, * *p* < 0.05, treatment groups vs. MOD. Table S8 Antibodies used for Western blot analysis (Further details).

## Abbreviations

The following abbreviations are used in this manuscript:

ABTS	2,2′-Azino-bis(3-ethylbenzothiazoline-6-sulfonic acid)
AP-1	Activator Protein-1
AQP-3	Aquaporin-3
CCK-8	Cell Counting Kit-8
CE	Cornified envelope
CTL	Control group
DH	Degree of hydrolysis
DPPH	2,2-Diphenyl-1-picrylhydrazyl
ECM	Extracellular matrix
EdU	5-Ethynyl-2′-deoxyuridine
ERK1/2	Extracellular signal-regulated kinase 1/2
FLG	Filaggrin
FRAP	Ferric-reducing antioxidant power
GAPDH	Glyceraldehyde-3-phosphate dehydrogenase
HA	Hyaluronic acid
HaCaT	Human immortalized keratinocyte cell line
HAS-2	Hyaluronan synthase -2
IL-1β	Interleukin-1 beta
IL-6	Interleukin-6
IL-8	Interleukin-8
INV	Involucrin
JNK	c-Jun N-terminal kinase
LOR	Loricrin
MAPK	Mitogen-Activated Protein Kinase
MDA	Malondialdehyde
MMP-1	Matrix metalloproteinase-1
MMP-3	Matrix metalloproteinase-3
MOD	Model group (UVB-exposed group)
NMFs	Natural moisturizing factors
NF-κB	Nuclear Factor Kappa B
PBS	Phosphate-buffered saline
ROS	Reactive Oxygen Species
RT-qPCR	Real-Time Quantitative Polymerase Chain Reaction
SDS-PAGE	Sodium dodecyl sulfate–polyacrylamide gel electrophoresis
SOD	Superoxide dismutase
T-AOC	Total antioxidant capacity
TD	Native *Dendrobium officinale* protein
TDM	Crude *Dendrobium officinale* protein enzymatic hydrolysate
TDM-5	*Dendrobium officinale* protein hydrolysate ultrafiltration fraction (10–50 kDa)
TDM-1	*Dendrobium officinale* protein hydrolysate ultrafiltration fraction (<10 kDa)
TGF-β	Transforming Growth Factor—βTNF-α Tumor Necrosis Factor—*α* UVB Ultraviolet B
WB	Western blot
