# Supplementary material for: Photoprotective Effects of Dendrobium officinale Protein Hydrolysate Fractions Against UVB-Induced Photoaging Associated with Modulation of MAPK/NF-κB and TGF-β/Smad Signaling
Source: Molecules. 2026 Jun 7;31(12):1990. doi: 10.3390/molecules31121990 (PMC13304510; doi:10.3390/molecules31121990)
Supplement: Supplementary file 1 [file molecules-31-01990-s001.zip › molecules-4286217-supplementary.pdf]

Supplementary Materials

Supplementary Methods

S1 Protein extraction

S1.1 Determination of Total Protein Content in Dendrobium officinale Powder by the Kjeldahl Method

Referring to the Kjeldahl method described in GB 5009.5–2016, the protein content in Dendrobium officinale powder was determined. Three portions of Dendrobium officinale powder (1.0 g each) were accurately weighed and transferred into 50 mL digestion tubes. Subsequently, 0.2 g of copper sulfate and 3.0 g of potassium sulfate were added as catalysts, followed by the addition of 10 mL of concentrated sulfuric acid for digestion to prepare the samples for subsequent nitrogen determination. After digestion, the tubes were removed and allowed to cool, and the volume was adjusted to 50 mL with deionized water. An aliquot of 5 mL from each tube was then introduced into an automatic Kjeldahl nitrogen analyzer for measurement, and three sets of experimental data were recorded. The total protein content was calculated according to Equation (S1).

$$X = \frac{(V_1 - V_2) \times 0.0140}{\left(m \times \frac{V_3}{100}\right)} \times F \times 10 \qquad (S1)$$

Where:
X: protein content, expressed in mg/g;
V_1: volume of sulfuric acid or hydrochloric acid titration solution consumed, in mL;
V_2: volume of sulfuric acid or hydrochloric acid titration solution consumed in the blank group, in mL;
0.0140: mass of nitrogen equivalent to 1 mL of 1 mol/L sulfuric acid or 1.0 mol/L hydrochloric acid standard titration solution, in g;
c: concentration of the sulfuric acid or hydrochloric acid titration solution, in mol/L;
m: mass of the sample, in g;
V_3: volume of the digested solution absorbed, in mL;
F: conversion factor from total nitrogen to protein; the nitrogen-to-protein conversion factor for *Dendrobium officinale* is 6.25;
10: conversion coefficient.

S1.2 Determination of Protein Extraction Concentration from *Dendrobium officinale*

Standard curve construction: The protein concentration was determined using a BCA Protein Assay Kit (Beyotime Biotechnology), and a standard curve was generated as shown in Equation (S2), with a coefficient of determination (R^2) of 0.9959.

$$y = 0.5349x - 0.1354 \quad (S2)$$

A protein standard stock solution (25 mg/mL) was prepared and diluted to 0.5 mg/mL to obtain the protein standard solution. Meanwhile, the BCA working reagent was prepared according to the instructions provided with the kit. Aliquots of 0, 1, 2, 4, 8, 12, 16, and 20 μL of the standard solution were sequentially added to the wells of a 96-well plate, and the volume was adjusted to 20 μL with the standard diluent. The corresponding standard protein concentrations were 0, 0.025, 0.05, 0.1, 0.2, 0.3, 0.4, and 0.5 mg/mL, respectively. Subsequently, 200 μL of BCA working reagent was added to each well, and the plate was incubated at 37 °C for 30 min. The absorbance was then measured at 562 nm using a microplate reader.

S1.3 Protein Extraction Process of *Dendrobium officinale*

Twenty grams of *Dendrobium officinale* powder was weighed and placed into a beaker. According to a solid–liquid ratio of 1:45, a preheated NaOH solution (0.075 mol/L) at 55 °C was added. The mixture was then placed in a water bath and maintained at 55 °C for extraction for 1 h, with continuous stirring to ensure thorough wetting and extraction.

After extraction, the residue was filtered through a 200-mesh nylon mesh, and the filtrate was retained. The pH of the filtrate was adjusted to 6.9 using 1 mol/L HCl. The filtrate was then stored in a refrigerator. During refrigeration, water-insoluble substances gradually flocculated. After flocculation was complete, the mixture was filtered to remove the precipitate, and the supernatant was collected to obtain a clear, light-yellow *Dendrobium officinale* protein solution without visible precipitation.

After obtaining the above protein solution, 1 mol/L HCl was further added to adjust the pH to 4.0. At this point, proteins near their isoelectric point precipitated. The mixture was centrifuged at 4000 r/min for 20 min, and the supernatant was discarded. Pure water was then added to the protein precipitate at a solid–liquid ratio of 1:3.5 (medicinal powder:water), and the pH of the solution was adjusted to 6.9. The protein concentration was subsequently determined according to the method described in Section S1.2.

The protein solution was then transferred into a 3 kDa dialysis bag and dialyzed against pure water under refrigerated conditions. The dialysis water was replaced every 3 h for a total of four times. Subsequently, the dialysis bag was transferred into deionized water and further dialyzed under refrigerated conditions for 12 h, during which the deionized water was replaced twice. After dialysis, the solution inside the dialysis bag was collected and filtered through a 0.45 μm microporous membrane to remove impurities, yielding a clear protein aqueous solution free of visible contaminants. Finally, the protein solution was freeze-dried to obtain *Dendrobium officinale* protein powder.

S1.4 Single-Factor Experiments for the Extraction of *Dendrobium officinale* Protein by the Alkali Extraction–Acid Precipitation Method

In the single-factor experiments, the effects of different parameters on the extraction yield of *Dendrobium officinale* protein were investigated. First, with the NaOH concentration fixed at 0.075 mol/L, extraction time at 1 h, and solid–liquid ratio at 1:45, the extraction temperatures were set at 45, 50, 55, 60, and 65 °C to evaluate the effect of temperature on protein extraction efficiency.

Subsequently, under the conditions of an extraction temperature of 55 °C, NaOH concentration of 0.075 mol/L, and solid–liquid ratio of 1:45, the extraction time was varied (0.5, 1, 1.5, 2, and 2.5 h) to investigate its effect on the extraction yield.

Next, with the extraction time fixed at 1 h, extraction temperature at 55 °C, and solid–liquid ratio at 1:45, different NaOH concentrations (0.025, 0.05, 0.075, 0.1, and 0.125 mol/L) were applied to explore the effect of alkali concentration on protein solubilization.

Finally, with the NaOH concentration maintained at 0.075 mol/L, extraction time at 1 h, and extraction temperature at 55 °C, the solid–liquid ratios were set at 1:25, 1:30, 1:35, 1:45, 1:55, and 1:65 (g/mL) to determine the optimal solid–liquid ratio.

All experiments were conducted in triplicate, and the average values were used for analysis.

S1.5 Protein Precipitation under Acidic Conditions

The *Dendrobium officinale* protein solution obtained under the extraction conditions described in Section S1.3 was selected. A 100 mL aliquot of the supernatant was taken, and the pH was adjusted to different values (3.0, 3.5, 4.0, 4.5, and 5.0) using 0.1 mol/L hydrochloric acid to induce protein precipitation. The sedimentation rate of the protein and the amount of flocculent precipitate formed were observed. The protein content in the extraction supernatant under different acidic pH conditions was determined according to the protein determination method described in Section S1.2. Each experiment was performed in triplicate.

S1.6 Statistical Analysis

Experimental data were statistically analyzed using SPSS and Origin 2021 software, and graphical representations were generated accordingly. The response surface experimental design was conducted using Design-Expert 13 software. All measurement results were expressed as mean ± standard deviation (mean ± SD), and analysis of variance (ANOVA) was performed to evaluate the significance of the effects of different factors on the response variables. Finally, the optimal extraction conditions determined from the analysis were validated by conducting three replicate experiments, and the protein extraction yield was

calculated to verify the predictive accuracy of the model and the stability of the method.

S2 Enzymatic hydrolysis

S2.1 Enzymatic Hydrolysis Conditions

A total of 0.15 g of *Dendrobium officinale* protein powder was weighed and dissolved in 50 mL of deionized water to prepare a protein solution with a substrate concentration of 0.3%. The pH of the protein solution was then adjusted to 6.9 using 0.1 mol/L HCl and 0.1 mol/L NaOH.

A compound protease preparation (papain: neutral protease = 1:1) was added to the protein solution at a dosage of 5000 U/g (2500 U papain and 2500 U neutral protease). The enzymatic hydrolysis reaction was carried out at an appropriate temperature using a temperature-controlled magnetic stirrer. After completion of hydrolysis, the enzyme was inactivated by heating the reaction mixture in a water bath at 90 °C for 15 min. The solution was then cooled to room temperature and centrifuged at 5000 rpm for 15 min, after which the supernatant was collected.

S2.2 Ultrafiltration Fractionation

The supernatant was divided into three portions and sequentially filtered through membranes with pore sizes or molecular weight cut-offs of 0.45 μm, 50 kDa, and 10 kDa, respectively. The filtrates were collected and subsequently freeze-dried to obtain *Dendrobium officinale* protein hydrolysates with different molecular weight ranges: TDM (total hydrolysate obtained after 0.45 μm filtration), TDM-5 (fraction with a molecular weight range of 50 kDa > TDM-5 > 10 kDa), and TDM-1 (fraction with a molecular weight <10 kDa).

S2.3 Freeze-Drying and Storage

The obtained filtrates were aliquoted into freeze-drying trays with the liquid layer thickness controlled to less than 1 cm, and then frozen in a −80 °C freezer until completely solidified. The freeze-dryer refrigeration system was started, and the cold trap was pre-cooled for 30 min. Once the temperature of the cold trap decreased to below −80 °C, the frozen samples were rapidly transferred into the freeze-dryer and the vacuum pump was activated. The cold trap temperature was maintained below −80 °C, and the system absolute pressure was kept below 1 Pa to allow sublimation drying for 48 h. After freeze-drying, the samples were immediately removed, sealed, and stored at −20 °C in the dark until use, while avoiding repeated freeze–thaw cycles.

S3 Degree of hydrolysis

S3.1 Determination of DH by the OPA Method

Preparation of OPA working solution: 3.810 g of sodium tetraborate and 0.1 mg of SDS (sodium dodecyl sulfate) were weighed and placed in a 150 mL beaker, and 75 mL of deionized water was added to dissolve them to obtain Solution A. Subsequently, 80 mg of o-phthalaldehyde was accurately weighed and dissolved in 2 mL of ethanol in the dark to obtain Solution B. Solution A and Solution B were mixed and transferred into a 100 mL volumetric flask. Then, 88 mg of DTT (dithiothreitol) was weighed and dissolved in the mixed solution. Finally, the solution was diluted to 100 mL with deionized water and stored in the dark to obtain the OPA working solution.

Preparation of standard solutions: 10 mg of serine powder was accurately weighed and dissolved in 100 mL of deionized water to obtain a 0.1 mg/mL serine stock solution. Aliquots of the standard solution (50, 100, 200, 300, and 400 μL) were transferred into 5 mL EP tubes, followed by the addition of 350, 300, 200, 100, and 0 μL of deionized water, respectively. Subsequently, 3 mL of OPA working solution was added to each EP tube, mixed thoroughly by vortexing, and allowed to react in the dark for 2 min. The absorbance was then measured at OD 340 nm. The amino acid concentrations and corresponding absorbance values were used to construct a standard curve, as shown in Equation (S3), with a coefficient of determination (R^2) of 0.9995.

$$y = 2.696x - 0.816 \qquad (S3)$$

Preparation of sample solutions: 30 mg of protein powder and enzymatic hydrolysate ultrafiltration product powder were accurately weighed, respectively, and dissolved in 10 mL of deionized water to obtain sample stock solutions with a concentration of 3 mg/mL. An aliquot of 200 μL of the sample stock solution was mixed with 200 μL of deionized water, followed by the addition of 3 mL of OPA working solution. The mixture was vortexed thoroughly in the dark and allowed to react for 2 min. The absorbance was then measured at 340 nm, and the degree of protein hydrolysis was calculated. The degree of hydrolysis was determined according to Equation (S4):

$$DH(\%) = \frac{(ht - hc)}{htot} \times DF \times 100\% \qquad (S4)$$

Where:

DH (%): degree of hydrolysis;

h_t: degree of hydrolysis of the protein relative to the original substrate after the hydrolysis reaction;

hc: degree of hydrolysis of the protein relative to the original substrate before the hydrolysis reaction;

DF: dilution factor;

h_{tot}: conversion factor for *Dendrobium officinale*, which is 8.

S3.2 Single-Factor Conditions for Enzymatic Hydrolysis of *Dendrobium officinale* Protein

With the substrate concentration fixed at 0.3%, enzymatic hydrolysis pH at 6.9, enzyme dosage at 5000 U, and hydrolysis temperature at 55 °C, the effect of hydrolysis time (1, 2, 3, 4, and 5 h) on the degree of hydrolysis of *Dendrobium officinale* protein (TD) was investigated.

With the substrate concentration fixed at 0.3%, hydrolysis time at 3 h, enzyme dosage at 5000 U, and hydrolysis temperature at 55 °C, the effect of pH values (5, 6, 7, 8, and 9) on the degree of protein hydrolysis was examined.

With the substrate concentration fixed at 0.3%, hydrolysis time at 3 h, hydrolysis pH at 6.9, and enzyme dosage at 5000 U, the effect of temperature (45, 50, 55, 60, and 65 °C) on the degree of protein hydrolysis was evaluated.

With the hydrolysis time fixed at 3 h, substrate concentration at 0.3%, hydrolysis pH at 6.9, and hydrolysis temperature at 55 °C, the effect of enzyme dosage (3000, 5000, 7000, 9000, and 11,000 U) on the degree of protein hydrolysis was investigated.

Each experiment was conducted in triplicate.

S3.3 Box–Behnken Design and Statistical Analysis

Based on the results of the single-factor experiments, a Box–Behnken design was employed for experimental design and response surface optimization to further optimize the enzymatic hydrolysis process of *Dendrobium officinale* protein. Three factors were selected for the experiment: hydrolysis time (A), enzyme dosage (B), and hydrolysis pH (C). Each factor was set at three levels, and the degree of protein hydrolysis was used as the response indicator. The specific experimental design is shown in Table S1.

S4 SDS-PAGE

S4.1 Preparation of SDS–PAGE Gels

(1) Preparation of 1× TBST buffer: 100 mL of 10× TBST buffer was diluted with purified water to a final volume of 1000 mL to obtain 1× TBST buffer.

(2) Preparation of 10× electrophoresis buffer: 30.3 g Tris, 144 g glycine, and 10.0 g SDS (sodium dodecyl sulfate) were added into a 2 L beaker and dissolved in 1 L of ultrapure water.

(3) Preparation of 1× electrophoresis buffer: 100 mL of 10× electrophoresis buffer was transferred into a graduated cylinder and diluted with ultrapure water to 1000 mL, followed by thorough mixing to obtain 1× electrophoresis buffer.

(4) Preparation of 10% separating gel: 2.7 mL distilled water, 3.3 mL 30% Acr-Bis solution (29:1), 3.8 mL 1 M Tris buffer (pH 8.8), and 0.1 mL 10% SDS solution were sequentially added into a test tube and mixed well. Subsequently, 0.1 mL of 10% ammonium persulfate (APS) and 0.004 mL TEMED were added, and the mixture was rapidly poured into the gel plates to complete polymerization.

(5) Preparation of 5% stacking gel: 2.1 mL distilled water, 0.5 mL 30% Acr-Bis solution (29:1), 0.38 mL 1 M Tris buffer (pH 6.8), and 0.03 mL 10% SDS were sequentially added and mixed. Then, 0.03 mL of 10% APS and 0.003 mL TEMED were added. After thorough mixing, the solution was layered onto the separating gel and allowed to polymerize completely.

(6) SDS–PAGE procedure: A 5% stacking gel and 10% separating gel were used to determine the molecular weights of TD and TDM. Briefly, 20 mg of TD and TDM freeze-dried powders were each dissolved in 10 mL of deionized water with shaking. After the foam dissipated, sample stock solutions with a concentration of 20 mg/mL were obtained. The stock solutions were diluted fourfold, and 10 μL of each diluted sample and 10 μL of protein marker (pre-stained protein marker, Cat. No. 26616, Thermo Fisher Scientific; molecular weight range 10–180 kDa) were loaded into the wells. Electrophoresis was first carried out at 80 mV for 30 min through the stacking gel, followed by 120 mV for 90 min through the separating gel. After electrophoresis, the gel was subjected to silver staining according to the procedure described in Section S4.3.

S4.2 Preparation of Silver Staining Solutions

(1) Preparation of fixation solution: 50 mL ethanol, 10 mL glacial acetic acid, and 40 mL deionized water were mixed thoroughly. This solution should be freshly prepared before use.

(2) Preparation of 30% ethanol solution: 30 mL absolute ethanol was mixed with 70 mL deionized water.

(3) Preparation of silver staining sensitizer working solution: 1 mL of silver staining sensitizer stock solution was mixed with 99 mL ultrapure water and vortexed for 1 min. The solution should be used within 2 h after preparation and stored at 4 °C in the dark.

(4) Preparation of silver ion working solution: 1 mL of silver solution was mixed with 99 mL deionized water and stored in the dark. The solution should be used within 2 h.

(5) Preparation of the color development system: For the basic developing solution, 20 mL of developing stock solution was mixed with 80 mL deionized water, followed by the addition of 50 μL formaldehyde solution. The mixture was thoroughly mixed and used within 3 min.

(6) Preparation of reaction termination solution: 5 mL of silver staining stop solution was mixed with 95 mL deionized water. The solution should be used on the same day of preparation.

S4.3 Silver Staining Procedure

(1) Fixation: After electrophoresis, the gel was transferred into 100 mL of fixation solution for fixation. The gel was placed on a shaker at room temperature (50–60 rpm) and continuously agitated for 40 min to remove residual electrophoresis reagents. Extending the fixation time can reduce background staining. After fixation, the solution was discarded.

(2) Pretreatment: The gel was washed with 100 mL of 30% ethanol for 10 min, followed by treatment with 200 mL of deionized water twice (first for 20 min and then for 1 min). The solution was then discarded. This pretreatment enhances the permeability of the gel matrix, facilitating the penetration of subsequent reagents into the gel.

(3) Sensitization: The gel was incubated with 100 mL of silver staining sensitizer solution for 2 min, followed by rapid washing with deionized water twice (1 min each time). The total washing time should be kept within 1.5 min to avoid residual sensitizer. Sensitization enhances staining sensitivity through thiosulfate-mediated complexation with silver ions.

(4) Silver ion binding: After washing, 100 mL of silver nitrate solution was added, and the gel was incubated in the dark for 5–10 min to allow specific binding of silver ions to the sulfhydryl and amino groups of proteins. The gel was then washed with deionized water for 1–1.5 min to remove unbound silver ions.

(5) Color development and termination: After washing, 100 mL of silver staining developing solution was added, and the color development process was monitored for 3–10 min. Once the target protein bands became clearly visible, the reaction was immediately terminated. The gel was treated with 100 mL of stop solution for 10 min to stabilize the staining result, followed by rinsing with deionized water for 2–5 min to complete the staining procedure. The gel was then scanned using a gel imaging system.

(6) Imaging: Distribution profile of *Dendrobium officinale* protein enzymatic hydrolysates. The stained gels were scanned using a gel documentation system.

S5 Antioxidant assays

S5.1 Determination of DPPH Radical Scavenging Activity of *Dendrobium officinale* Protein Hydrolysates

Twelve milligrams of DPPH powder was weighed and dissolved in 100 mL of absolute ethanol, followed by ultrasonic dissolution for 30 min to prepare the DPPH radical working solution. As DPPH radicals are photosensitive and susceptible to environmental oxidation, the DPPH working solution should be freshly prepared before use.

Then, 100μL of the sample solution was added into a well of a 96-well plate, followed by the addition of 100μL of DPPH working solution. The mixture was allowed to react at room temperature for 30 min, and the absorbance (A_x) was measured at 517 nm using a microplate reader. The absorbance of a mixture of 100μL sample solution and 100μL absolute ethanol was measured as A_0, while the absorbance of a mixture of 100μL deionized water and 100μL DPPH working solution was recorded as A_1. Each experiment was performed in triplicate. The DPPH radical scavenging rate was calculated according to Equation (S5).

$$\text{DPPH radical scavenging rate\%} = \left(1 - \frac{A_X - A_0}{A_1}\right) \times 100\% \quad \text{(S5)}$$

Where:

A_x: absorbance of the mixture containing 100μL sample solution and 100μL DPPH working solution;

A_0: absorbance of the mixture containing 100μL sample solution and 100μL absolute ethanol;

A_1: absorbance of the mixture containing 100μL deionized water and 100μL DPPH working solution.

S5.2 Determination of ABTS Radical Scavenging Activity of *Dendrobium officinale* Protein Hydrolysates

Potassium persulfate solution (Solution A) was prepared by dissolving 3.78 g of potassium persulfate in 10 mL of deionized water to obtain a concentration of 0.0378 g/mL. ABTS solution (Solution B) was prepared by dissolving 0.384 g of ABTS reagent in 10 mL of deionized water to obtain a concentration of 0.00384 g/mL. Subsequently, 0.088 mL of Solution A was mixed with 5 mL of Solution B and stored overnight at room temperature in the dark to obtain the ABTS stock solution.

Before use, the ABTS stock solution was diluted with deionized water until the absorbance at 734 nm reached 0.70 ± 0.02, yielding the ABTS working solution. Then, 10 μL of TD, TDM, TDM-5, and TDM-1 sample solutions at different concentrations were added into the wells of a 96-well plate, followed by the addition of 190 μL of ABTS working solution. The mixture was gently mixed and incubated in the dark for 10 min, after which the absorbance was measured at 734 nm using a microplate reader. The ABTS radical scavenging rate was calculated according to Equation (S6). Each experiment was performed in triplicate.

$$\text{ABTS radical scavenging rate\%} = \left(1 - \frac{A_t - A_r}{A_0}\right) \times 100\% \quad \text{(S6)}$$

Where:

A_t: absorbance of the mixture containing 10μL sample solution and 190μL ABTS working solution;

A_r: absorbance of the mixture containing 10μL sample solution and 190μL deionized water;

A_0: absorbance of the mixture containing 10μL deionized water and 190μL ABTS working solution.

S5.3 Determination of FRAP Total Antioxidant Capacity of *Dendrobium officinale* Protein Hydrolysates

S5.3.1 Construction of the Standard Curve for Ferric Ion Reducing Power

The required reagent solutions were prepared, including 0.2 mol/L PBS (pH 6.6), 1% potassium ferricyanide solution, 10% trichloroacetic acid ($C_2HCl_3O_2$), and 0.1% ferric chloride solution. In addition, 0.278 g of $FeSO_4{\cdot}7H_2O$ was accurately weighed, dissolved in a 10 mL volumetric flask, and diluted to volume to obtain a 100 mmol/L Fe^{2+} standard solution. This solution was further diluted to prepare a series of concentration gradients (0.15, 0.3, 0.6, 0.9, 1.2, and 1.5 mmol/L) for construction of the standard curve.

During the determination, 100 μL of sample solution, 100 μL of phosphate buffer solution, and 100 μL of potassium ferricyanide solution were mixed and incubated in a 50 °C water bath for 20 min. Subsequently, 100 μL of trichloroacetic acid was added to terminate the reaction. Then, 100 μL of the reaction mixture was taken and mixed with 100 μL of deionized water and 20 μL of ferric chloride solution. After reacting in the dark at room temperature for 10 min, the absorbance was measured at 700 nm. The obtained standard curve is shown in Equation (S7), with a determination coefficient of R^2= 0.9991. The ferric reducing antioxidant power (FRAP value) of the samples was calculated according to the standard curve.

$$y = 1.4179X - 0.0048 \qquad (S7)$$

S5.3.2 Determination of Ferric Ion Reducing Power of the Samples

Briefly, 100 μL of the sample solution, 100 μL of 0.2 mol/L phosphate buffer solution, and 100 μL of 1% potassium ferricyanide solution were sequentially mixed thoroughly and incubated in a constant-temperature water bath at 50 °C for 20 min. Subsequently, 100 μL of 10% trichloroacetic acid was added to terminate the reaction.

Then, 100 μL of the reaction mixture was transferred into a well of a 96-well plate, followed by the addition of 100 μL deionized water and 20 μL of 0.1% $FeCl_3$ solution, and allowed to react for 10 min. The absorbance was measured at 700 nm. The absorbance value was substituted into Equation (S7) to calculate the antioxidant capacity. Each experiment was performed in triplicate.

S5.4 Determination of Hydroxyl Radical Scavenging Activity of *Dendrobium officinale* Protein Hydrolysates

Salicylic acid solution (5 mmol/L) was prepared by dissolving 0.06906 g of salicylic acid in 100 mL of deionized water. A 5 mmol/L $FeSO_4$ solution was prepared by dissolving 0.076 g of $FeSO_4$ in a volumetric flask and diluting to 100 mL with deionized water. In addition, 0.57 mL of

hydrogen peroxide (H_2O_2) solution was diluted to 100 mL with deionized water to obtain a 5 mmol/L H_2O_2 working solution.

Then, 100 μL of sample solution was transferred into an EP tube, followed by the sequential addition of 200 μL salicylic acid solution, 200 μL $FeSO_4$ solution, and 100 μL H_2O_2 solution. After thorough mixing, the reaction mixture was incubated in a water bath at 37 °C for 1 h. After completion of the reaction, 200 μL of the reaction solution was transferred to a 96-well plate, and the absorbance (A_x) was measured at 517 nm using a microplate reader.

For the blank control, deionized water was used instead of H_2O_2, and the absorbance was recorded as A_0. For the negative control, deionized water was used instead of the sample solution, and the absorbance was recorded as A_1. The hydroxyl radical scavenging rate was calculated according to Equation (S8). Each experiment was performed in triplicate.

$$\text{Hydroxyl Radical Scavenging Activity\%} = \left(1 - \frac{A_X - A_0}{A_1}\right) \times 100\% \qquad \text{(S8)}$$

Where:

A_x: absorbance of the mixture containing 100 μL sample solution + 200 μL salicylic acid solution + 200 μL $FeSO_4$ solution + 100 μL H_2O_2 solution.

A_0: absorbance of the mixture containing 100 μL sample solution + 200 μL salicylic acid solution + 200 μL $FeSO_4$ solution + 100 μL deionized water.

A_1: absorbance of the mixture containing 100 μL deionized water + 200 μL salicylic acid solution + 200 μL $FeSO_4$ solution + 100 μL H_2O_2 solution.

S5.5 Statistical analysis

All assays in this study were performed in at least triplicate. Data processing and graphical presentation were conducted using SPSS and Design-Expert 13 software. The experimental results are expressed as mean ± standard deviation (SD), and analysis of variance (ANOVA) was performed to evaluate statistical differences.

S6 Cell experiments

S6.1 Cell Treatment and UVB Irradiation

The HaCaT cell line was obtained from Shanghai Fuheng Biotechnology Co., Ltd. *Dendrobium officinale* protein TD and its enzymatic hydrolysis ultrafiltration fractions (TDM, TDM-5, and TDM-1) were used as experimental materials. Primers for Filaggrin, IL-1β, and MMP-1 were synthesized by Sangon Biotech (Shanghai) Co.

S6.1.1 Preparation of Culture Medium and Drug Solutions

Complete culture medium:10 mL of fetal bovine serum and 1 mL of penicillin–streptomycin mixture were added to 90 mL of DMEM medium, mixed thoroughly, and stored at 4 °C under refrigeration.

Preparation of sample solutions: *Dendrobium officinale* protein enzymatic hydrolysate and its ultrafiltration fractions (TD, TDM, TDM-5, and TDM-1) were accurately weighed (50 mg each) and dissolved in separate centrifuge tubes. Subsequently, 5 mL of deionized water was added to each tube and mixed thoroughly. The solutions were sterilized through a 0.22 μm microporous membrane to obtain sample solutions with a concentration of 10 mg·mL^{-1}.

Preparation of the positive control:10 mg of ascorbic acid powder was accurately weighed and dissolved in 10 mL of deionized water. The solution was protected from light and mixed until completely dissolved, followed by sterilization through a 0.22μm microporous membrane to obtain a stock solution with a concentration of 1 mg·mL^{-1}.

S6.1.2 Thawing, Passaging, and Cryopreservation of HaCaT Cells

Cell thawing:The water bath was preheated to 37 °C. HaCaT cryopreserved cells stored at −80 °C were removed and immediately placed in the water bath for thawing, with gentle agitation to facilitate melting. The thawed cell suspension was then carefully transferred into a 15 mL centrifuge tube, followed by the addition of 5 mL DMEM medium and gentle pipetting to mix. The suspension was centrifuged at 1000 rpm for 3 min, and the supernatant was discarded. The cell pellet was resuspended in 1 mL complete culture medium. Finally, the cell suspension was transferred into a culture flask containing 4 mL complete medium, gently mixed, and incubated in a humidified incubator at 37 °C with 5% CO_2.

Cell passaging: HaCaT cells were cultured in complete DMEM medium supplemented with 10% fetal bovine serum and 1% penicillin–streptomycin, and maintained in a humidified incubator at 37 °C with 5% CO_2. When the cell confluence reached approximately 85%, passaging was performed. The culture medium was first removed, and the cells were rinsed with PBS buffer to eliminate residual serum components, repeating the wash 2–3 times. Subsequently, 1 mL of 0.25% trypsin was added for cell digestion. After approximately 1 min, when the intercellular spaces enlarged and cells began to detach under microscopic observation, the trypsin solution was immediately discarded, and 2 mL complete medium was added to terminate the digestion. The cells were then resuspended and transferred into a 15 mL centrifuge tube, followed by centrifugation at 1000 rpm for 3 min. After discarding the supernatant, 15 mL complete medium was added to resuspend the cells. Finally, the cell suspension was evenly distributed into three new culture flasks at a ratio of 1:3 and incubated for further subculture.

Cell cryopreservation: HaCaT cells in the logarithmic growth phase were selected. The old culture medium was discarded, and the cells were gently rinsed twice with PBS buffer. Then, 1 mL of 0.25% trypsin was added for digestion at 37 °C for approximately 1 min. When enlarged intercellular spaces were observed, the trypsin solution was discarded immediately, and complete medium was added to terminate digestion to prevent cell damage caused by over-digestion. The cell suspension was then transferred into a centrifuge tube and centrifuged at 1000 rpm for 3 min. After removing the supernatant, 1 mL of cryopreservation solution was added and mixed thoroughly. The cells were placed in a programmed freezing container for

pre-cooling and finally transferred to a −80 °C freezer for short-term storage.

S6.1.3 Cell Seeding and Grouping

The HaCaT cell culture flasks in the incubator were removed, and when the cells reached 80%–90% confluence, 0.25% trypsin was added for digestion to prepare a cell suspension. The cells were counted under a microscope and then seeded into 96-well plates (5×10^4 cells/well) or 6-well plates (7.5×10^5 cells/well). According to the experimental conditions described in Section S6.1.6, a UVB-induced photoaging injury model of HaCaT cells was established.

S6.1.4 Determination of HaCaT Cell Viability by CCK-8 Assay

HaCaT cells were seeded into 96-well plates, with 100μL of complete medium added to each well, and cultured in an incubator at 37 °C with 5% CO_2. The cells were then grouped and treated with the corresponding drugs, with six replicate wells in each group. After model establishment and drug treatment, CCK-8 reagent was mixed with culture medium at a ratio of 1:10 to prepare the CCK-8 working solution. The original medium was discarded and the wells were gently rinsed with PBS. Subsequently, 100 μL of CCK-8 working solution was added to each well. A blank control group was also set up, containing 100 μL of CCK-8 working solution without cells. After incubation in the incubator for 40 min, the plates were measured using a microplate reader at 450 nm to determine the optical density (OD) values.

Cell viability (%) = (OD_ experimental − OD_ blank) / (OD_ control − OD_ blank) × 100%

S6.1.5 Cytotoxic Effects of TD, TDM, TDM-5, and TDM-1 on HaCaT Cells

HaCaT cells were seeded into four 96-well plates at a density of 5×10^4 cells per well. The plates were grouped according to the experimental materials, with each plate serving as one treatment group. Different concentrations (100, 200, 400, and 800 μg/mL) of TD, TDM, TDM-5, and TDM-1 were prepared in complete culture medium and added to the corresponding wells to establish concentration gradients. The treated plates were incubated at 37 °C in a 5% CO_2 incubator and cultured for 12, 24, and 48 h after treatment. At the end of each designated time point, the supernatant medium was discarded, and the absorbance values of each well were measured according to the method described in **S6.1.4**, from which cell viability was calculated.

S6.1.6 Establishment of a UVB-Induced Photoaging Damage Model in HaCaT Cells

According to the method described in S6.1.3, HaCaT cells were seeded into 96-well plates. After cell attachment, the culture medium was discarded and the cells were washed three times with PBS. The plates were then exposed to UVB irradiation using a UVB lamp (15 W, 280–380 nm, peak at 315 nm) positioned approximately 10 cm above the 96-well plate. The UV intensity was measured at three different positions to ensure uniform irradiation. Based on the formula

irradiation dose = *UVB irradiation power* × *exposure time*, different irradiation dose gradients were set at 0, 10, 20, 40, 60, and 80 mJ·cm^{-2}.

After irradiation, the cells were further cultured for 12 h and 24 h. The OD values of each well were measured according to the method described in S6.1.4, and cell viability was calculated. Meanwhile, following the procedure described in S6.1.8, intracellular reactive oxygen species (ROS) levels were determined by flow cytometry, and the mRNA expression level of the keratinization barrier factor filaggrin (FLG) was analyzed using RT-qPCR. By comprehensively analyzing these indicators, the optimal UVB dose was selected to establish a stable UVB-induced photoaging damage model in HaCaT cells.

S6.1.7 Determination of the Effects of Different Fractions on the Proliferation of UVB-Induced HaCaT Cells by EdU Labeling

HaCaT cells in the control group (CTL), model group (MOD), positive control group (ascorbic acid), and each experimental treatment group were seeded into 6-well plates at a density of 7.5 × 10^5 cells per well. The photoaging damage model was established according to the procedure described in **S6.1.6**, and the experimental grouping was set as described in **S6.1.3**. After cell attachment, the corresponding intervention drugs were added to each group and incubated for 12 h. Following treatment, the culture supernatant was discarded, and the cells were gently washed with an appropriate amount of PBS. Cell proliferation in each group was then observed under a microscope.

S6.1.8 Determination of Intracellular ROS Levels in HaCaT Cells After UVB Irradiation

Intracellular ROS levels in HaCaT cells were measured using flow cytometry according to the instructions of the reactive oxygen species detection kit. First, HaCaT cells in the logarithmic growth phase were seeded into 6-well plates. Modeling and grouping were performed according to the procedures described in S6.1.6 and S6.1.3, respectively. After 12 h of intervention, the culture medium was discarded and the cells were washed three times with PBS. The cells were then collected and incubated with serum-free culture medium containing 10 μmol/L probe at 37 °C in the dark for 20 min. After incubation, the cells were washed three times with PBS. Intracellular ROS levels were subsequently determined using a flow cytometer. The probe used was DCFH-DA (10 μM), with an excitation wavelength of 488 nm and detection in the FITC channel. A total of 1 × 10^6 cells were collected for analysis.

S6.1.9 Determination of Intracellular MDA, SOD, and T-AOC

The levels of antioxidant-related indicators in HaCaT cells after UVB irradiation were determined using commercial assay kits for MDA, SOD, and T-AOC according to the manufacturers' instructions. HaCaT cells were first seeded into 6-well plates at a density of 7.5 × 10^5 cells per well. The UVB-induced photoaging model was established according to the

method described in **S6.1.6**, and grouping was performed as described in **S6.1.3**. After treatment with the corresponding drugs for 12 h, the cells were washed 2–3 times with PBS. The cell samples were then collected, and the levels of MDA, SOD, and T-AOC in HaCaT cells after modeling and drug treatment were measured following the kit protocols. The therapeutic effects of each fraction were subsequently evaluated by analyzing the cellular oxidative stress status and antioxidant capacity.

S6.1.10 Detection of the Barrier Factor Filaggrin (FLG) mRNA in HaCaT Cells

S6.1.10.1 Extraction of Total RNA

HaCaT cells in the logarithmic growth phase were first seeded into 6-well plates. The photoaging model was established according to **S5.2.6**, and grouping was performed following **S5.2.3**. After 12 h of intervention, the culture medium was discarded and the cells were washed three times with PBS. Subsequently, 1 mL of Trizol lysis buffer was added to each well, and the lysate was transferred into RNase-free centrifuge tubes. Then, 200 μL chloroform was added and mixed thoroughly. The mixture was centrifuged at 11,000 g for 10 min at 4 °C in a pre-cooled high-speed centrifuge.

The supernatant was carefully transferred into a new RNase-free centrifuge tube, followed by the addition of an equal volume of isopropanol. After thorough mixing, the sample was placed in a pre-cooled high-speed centrifuge and centrifuged at 11,000 g for 10 min at 4 °C after standing for 2 min. The supernatant was discarded, and the resulting precipitate represented total RNA. The RNA pellet was then washed with pre-cooled 75% ethanol and centrifuged at 7,500 g for 5 min at 4 °C. After removing the supernatant, the RNA pellet was air-dried at room temperature, avoiding excessive drying. Finally, 10–40 μL RNase-free water was added to dissolve the RNA pellet, and the solution was incubated on ice for 30 min to allow complete re-dissolution and extension of the RNA.

S6.1.10.2 Determination of RNA Concentration

The concentration of RNA samples was determined using a NanoDrop spectrophotometer. RNase-free water was used as the blank for calibration. Subsequently, 1 μL of the RNA solution was loaded for measurement, and the purity and concentration were recorded.

S6.1.10.3 Reverse Transcription

Reverse transcription was performed using the extracted total RNA according to the instructions of the HyperScript™ First-Strand cDNA Synthesis SuperMix kit to synthesize first-strand cDNA for subsequent experiments.

S6.1.10.4 Primer Design

Target genes were selected from the NCBI database, and primers were designed using Primer 6.0 software. All primers were synthesized by Sangon Biotech (Shanghai) Co., Ltd. The qPCR primer sequences are listed as follows:

Table S1. RT-PCR Filaggrin primer sequences.

Gene	Sequence（5′-3′）
Filaggrin	F: ATCTGAGGGCACTGAAAGGC
Filaggrin	R: CACTTCCGTGCTGAGAGTGT
GAPDH	F: AATGGGCAGCCGTTAGGAAA
GAPDH	R: GCGCCCAATACGACCAAATC

S6.1.10.5 PCR Amplification of Target Genes

The reaction system was prepared according to the instructions of the HotStart™ 2× SYBR Green qPCR Master Mix kit, and all procedures were performed on ice to ensure reaction stability. Three replicate wells were set for each sample to ensure data accuracy. After completion of the amplification program, Ct values were collected using a real-time quantitative PCR instrument, and the mRNA expression levels were calculated.

S6.1.11 Detection of IL-1β Transcription Levels in HaCaT Cells

Following the experimental design described in S6.1.10, the target gene was selected from the NCBI database, and primers were designed using Primer 6.0 software. The IL-1β primers were synthesized by Bioengineering (Shanghai) Co., Ltd., with the sequences as follows:

Table S2. IL-1β Gene Primer Sequences.

Gene	Sequence（5′-3′）
IL-1β-F	F: AACCTCTTCGAGGCACAAGG
IL-1β-R	R: AGCCATCATTTCACTGGCGA
GAPDH-F	F: AATGGGCAGCCGTTAGGAAA
GAPDH-R	R: GCGCCCAATACGACCAAATC

S6.1.12 Detection of MMP-1 mRNA in HaCaT Cells

Following the experimental design described in S6.1.10, the target gene was selected from the NCBI database, and primers were designed using Primer 6.0 software. The MMP-1 primers were synthesized by Bioengineering (Shanghai) Co., Ltd., with the sequences as follows:

Table S3. MMP-1 gene primer sequences.

Gene	Sequence（5′-3′）
MMP-1	F: AGAAAGAAGACAAAGGCAAGTTGA
MMP-1	R: GCATGGTCCACATCTGCTCT
GAPDH	F: AATGGGCAGCCGTTAGGAAA
GAPDH	R: GCGCCCAATACGACCAAATC

S6.2 Preparation of SDS-PAGE Gel Solutions

According to the molecular weight of the cellular proteins, SDS-PAGE gels of appropriate concentrations were prepared. The required volumes of resolving gel were configured following the manufacturer's concentration ratios and thoroughly mixed. After pouring the resolving gel, a layer of anhydrous ethanol was overlaid to create a flat interface. The gel was left to stand for 35 min; the appearance of a distinct interface between the ethanol and the resolving gel indicated complete polymerization. The ethanol layer was then removed, and any residual liquid was absorbed with filter paper. Subsequently, the stacking gel was prepared according to the manufacturer's recommended concentration ratios, poured on top, and a comb was inserted. The gel was allowed to polymerize for an additional 35 min. Once fully set, the gel plates were mounted onto the apparatus and placed into the electrophoresis tank, ready for subsequent experiments.

S6.3 Determination of Sample Protein Content

Electrophoresis buffer was added to the tank, ensuring that the buffer level completely covered the sample wells. Samples were loaded sequentially according to the predetermined order, with 10 μL of each sample added per well. In the protein marker wells, 5 μL and 2 μL of protein marker were loaded, respectively, to facilitate molecular weight comparison.

S6.4 SDS-PAGE Electrophoresis

After connecting the electrophoresis apparatus, 1× running buffer was added to the tank. The comb was carefully removed, and samples were loaded into the wells. The voltage was initially set to 80 V for 20 min to allow the bromophenol blue tracking dye to compress into a sharp line and enter the resolving gel. The voltage was then increased to 200 V for 20 min, and electrophoresis was stopped once the bromophenol blue dye reached the front edge of the gel.

S6.5 Protein Transfer (Blotting)

After electrophoresis, the PVDF membrane was trimmed to the appropriate size and activated by soaking in methanol. The wet transfer method was employed, assembling the sandwich in the order: filter paper – gel – PVDF membrane – filter paper. The assembly was placed vertically in the transfer tank, which was filled with pre-chilled (4 °C) transfer buffer. Ice was

placed around the tank to maintain a low temperature. The power supply was connected, and the current was set to 400 mA for 300 min to complete the protein transfer.

S6.6 Blocking

After completing the protein transfer, the sandwich assembly was disassembled, and the PVDF membrane was removed. The membrane was rinsed in a container with 1× TBST three times on a shaker at 120 rpm for 8 min each. Subsequently, the membrane was incubated in rapid blocking solution on a shaker at 75 rpm for 10 min at room temperature. After blocking, the PVDF membrane was transferred back to a container with 1× TBST and washed three times on a shaker at 120 rpm for 8 min each.

S6.7 Primary Antibody Incubation

The washed PVDF membrane was removed and incubated with the target primary antibody diluted in primary antibody diluent according to the target protein. The membrane was incubated overnight at 4 °C.

S6.8 Secondary Antibody Incubation

After completion of primary antibody incubation, the PVDF membrane was removed and washed three times with 1×TBST. The washed membrane was then incubated in the secondary antibody solution on a shaker at 75 rpm for 2 h. Following incubation, the membrane was washed three times with 1×TBST, with each wash performed on a shaker at 120 rpm for 7 min.

S6.9 Visualization and Imaging

The chemiluminescent detection solution was prepared according to the manufacturer's instructions at a 1:1 ratio. The PVDF membrane was immersed in the detection solution for 1 min, then transferred to the imaging device and gently agitated to ensure uniform coverage. Band intensity was quantified using ImageJ software to calculate protein expression levels.

Supplementary Tables

Table S4. Response surface methodology design and analysis for protein extraction.

Factor	Level		
	-1	0	1
A(Temperature/℃)	55	60	65
B(Time/h)	1	1.5	2
C(Concentration mol/L)	0.075	0.1	0.125

(A)Factor levels used in RSM design

Experimental Group	Experimental Factor			Extraction Rate (%)
	A Temperature (℃)	B Time（h）	C Concentration (mol/L)	
1	65	1.5	0.125	34.06
2	65	1	0.1	34.16
3	60	2	0.125	34.57
4	60	1.5	0.1	41.01
5	60	1.5	0.1	40.94
6	55	1	0.1	34.27
7	60	1	0.075	26.44
8	60	2	0.075	27.68
9	55	1.5	0.125	35.04
10	65	1.5	0.075	28.55
11	60	1.5	0.1	39.76
12	55	1.5	0.075	27.12
13	60	1.5	0.1	41.69
14	60	1.5	0.1	40.97
15	60	1	0.125	31.26
16	65	2	0.1	35.59
17	55	2	0.1	32.41

(B)Box–Behnken design matrix and experimental results

Source of variance	Sum of squares	Degrees of freedom	Mean square	F-value	P-value	Significance
Model	403.86	9	44.87	55.82	<0.0001	Highly significant
A	1.64	1	1.64	2.04	0.1965	Not significant
B	2.02	1	2.02	2.51	0.1569	Not significant
C	79.00	1	79.00	98.27	<0.0001	Highly significant
AB	2.54	1	2.54	3.16	0.1085	Not significant
AC	1.45	1	1.45	1.81	0.2209	Not significant

Source of variance	Sum of squares	Degrees of freedom	Mean square	F-value	P-value	Significance
BC	1.07	1	1.07	1.33	0.2862	Not significant
A^2	29.98	1	29.98	37.29	0.0005	Significant
B^2	63.17	1	63.17	78.57	< 0.0001	Highly significant
C^2	195.45	1	195.45	243.13	< 0.0001	Highly significant
Residual	5.63	7	0.8039			
Lack of Fit Test	4.52	3	1.51	5.45	0.0676	not significant
Pure Error	1.11	4	0.2767			
Lack of Fit SS	409.49	16				

(C)ANOVA for regression model

Table S5. Optimization of protein hydrolysis using response surface methodology.

Factor	Level		
	-1	0	1
A (Enzymatic digestion time/ h)	2	3	4
B (Enzyme dosage/U)	5000	7000	9000
C (Hydrolysis pH)	6	7	8

(A) Factor levels

RUN	Factor			Hydrolysis Degree (%)
	A Enzymatic Digestion Time (h)	B Enzyme Dosage (U)	C Enzymatic Digestion pH	
1	3	7000	7	40.68
2	2	9000	7	32.08
3	4	7000	8	36.13
4	3	7000	7	40.46
5	3	7000	7	40.48
6	2	7000	8	39.28
7	4	7000	6	35.17
8	3	9000	8	37.05
9	3	5000	8	33.40

RUN	Factor			Hydrolysis Degree (%)
	A Enzymatic Digestion Time (h)	B Enzyme Dosage (U)	C Enzymatic Digestion pH	
10	2	7000	6	34.7
11	3	5000	6	33.82
12	3	9000	6	31.6
13	3	7000	7	40.88
14	2	5000	7	34.77
15	4	5000	7	31.06
16	3	7000	7	40.72
17	4	9000	7	33.21

(B) Experimental design and results

Regression equation:

$y = 40.64 - 0.6582A + 0.1113B + 1.32C + 1.21AB - 0.9075AC + 1.47BC - 2.75A^2 - 5.11B^2 - 1.57C^2$

Source of variance	Sum of squares	Degrees of freedom	Mean square	F-value	P-value	Significance
Model	200.85	9	22.32	242.60	<0.0001	Highly significant
A	3.47	1	3.47	37.74	0.0005	Significant
B	0.0990	1	0.0990	1.08	0.3340	Not significant
C	13.99	1	13.99	152.10	<0.0001	Highly significant
AB	5.86	1	5.86	63.66	<0.0001	Highly significant
AC	3.29	1	3.29	35.81	0.0006	Significant
BC	8.61	1	8.61	93.64	<0.0001	Highly significant
A^2	31.95	1	31.95	342.27	<0.0001	Highly significant
B^2	109.92	1	109.92	1194.93	< 0.0001	Highly significant
C^2	10.34	1	10.34	112.39	< 0.0001	Highly significant
Residual	0.6439	7	0.0920			

Source of variance	Sum of squares	Degrees of freedom	Mean square	F-value	P-value	Significance
Lack of Fit Test	0.5204	3	0.1735	5.62	0.0644	not significant
Pure Error	0.1235	4	0.0309			
Lack of Fit SS	201.50	16				

(C) Regression model and ANOVA

Data Analysis

Experimental data were processed and plotted using SPSS and Origin 2021, while the response surface methodology was designed and analyzed using Design-Expert 13. The experimental results were expressed as mean ± standard deviation (mean ± SD), and statistical significance was evaluated using analysis of variance (ANOVA).

To verify the optimization results, the experiment was repeated three times under the optimal hydrolysis conditions to determine the degree of hydrolysis of *Dendrobium officinale* protein, and the measured values were compared with the model-predicted values for validation.

Table S6

Table S6. Primer sequences used for RT-qPCR.

Gene	Forward (5′–3′)	Reverse (5′–3′)
FLG	ATCTGAGGGCACTGAAAGGC	CACTTCCGTGCTGAGAGTGT
IL-1β	AACCTCTTCGAGGCACAAGG	AGCCATCATTTCACTGGCGA
MMP-1	AGAAAGAAGACAAAGGCAAGTTGA	GCATGGTCCACATCTGCTCT
GAPDH	AATGGGCAGCCGTTAGGAAA	GCGCCCAATACGACCAAATC

Table S7

Table S7. Effects of TD, TDM, TDM-5, and TDM-1 on MDA, SOD, and T-AOC Levels in HaCaT Cells After UVB Irradiation. Ascorbic acid:5 $\mu g \cdot mL^{-1}$. ### $p < 0.001$, MOD vs CTL; *** $p < 0.001$, ** $p < 0.01$, * $p < 0.05$, treatment groups vs. MOD.

Experimental Groups	MDA ($nmol \cdot mg^{-1}$ protein)	SOD ($U/mgprot^{-1}$)	T-AOC ($mmol/mgprot^{-1}$)
CTL	5.11±0.93	2.15±0.11	2.56±0.04
MOD	18.21±0.23 (###)	0.35±0.11 (###)	0.76±0.11 (###)
Ascorbic acid	8.26±0.88 (***)	1.9±0.23 (***)	2.07±0.07 (***)

Experimental Groups	MDA (nmol·mg⁻¹ protein)	SOD (U/mgprot⁻¹)	T-AOC (mmol/mgprot⁻¹)
(5μg·mL⁻¹)			
TD (50 μg·mL⁻¹)	16.65±1.25	0.35±0.11	0.75±0.04
TD (100 μg·mL⁻¹)	14.18±0.77 (*)	0.56±0.11 (*)	0.83±0.03 (*)
TD (200 μg·mL⁻¹)	17.3±1.18	0.3±0.12	0.7±0.05
TDM (50 μg·mL⁻¹)	15.68±0.96	0.61±0.19 (**)	0.91±0.06 (*)
TDM (100 μg·mL⁻¹)	13.34±0.71 (**)	0.29±0.1	0.85±0.03 (**)
TDM (200 μg·mL⁻¹)	15.68±0.73	0.28±0.1	1.03±0.04 (**)
TDM-5 (50 μg·mL⁻¹)	14.16±1.4 (*)	1.05±0.11 (**)	0.99±0.05 (**)
TDM-5 (100 μg·mL⁻¹)	12.7±0.6 (*)	0.96±0.13 (**)	1.16±0.01 (**)
TDM-5 (200 μg·mL⁻¹)	10.85±0.16 (***)	0.92±0.12 (**)	1.3±0.02 (**)
TDM-1 (50 μg·mL⁻¹)	10.76±1.08 (***)	0.94±0.17 (**)	0.02±1.1 (**)
TDM-1 (100 μg·mL⁻¹)	7.8±0.68 (***)	1.39±0.08 (**)	1.66±0.04 (***)
TDM-1 (200 μg·mL⁻¹)	5.71±0.34 (***)	1.88±0.18 (**)	1.77±0.05 (**)

Table S8

Table S8. Antibodies used for Western blot analysis (Further details).

Antibody	Catalogue Number	Company
Phospho-p38Antibody	36-8500	Thermo Fisher Scientific, USA
Phospho-SAPK/JNK Antibody	44-682G	Thermo Fisher Scientific, USA.
Phospho-c-jun Antibody	PA5-17261	Thermo Fisher Scientific, USA.
Phospho-c-fos Antibody	PA5-36769	Thermo Fisher Scientific, USA.
Phospho-p65NF-κB Antibody	51-0500	Thermo Fisher Scientific, USA.
IκBα Antibody	39-7700	Thermo Fisher Scientific, USA.
Phospho-IκBα Antibody	12-9035-42	Thermo Fisher Scientific, USA.
p38 MAPK Antibody	AHO1202	Thermo Fisher Scientific, USA.
SAPK/JNK Antibody	35-9800	Thermo Fisher Scientific, USA.
c-jun Antibody	24909-1-AP	ProteinTech Group
c-fos Antibody	MA5-15055	Thermo Fisher Scientific, USA.
Phospho-Erk1/2 Antibody	17-9109-42	Thermo Fisher Scientific, USA.
Erk1/2 Antibody	13-6200	Thermo Fisher Scientific, USA.

Antibody	Catalogue Number	Company
TGF-β Antibody	MA5-15065	Thermo Fisher Scientific, USA.
Phspho-Smad2/3 Antibody	PA5-99378	Thermo Fisher Scientific, USA.
Smad-4 Antibody	MA5-15682	Thermo Fisher Scientific, USA.
Smad-7 Antibody	25850-1-AP	ProteinTech Group
